# PE/PPE proteins contribute to *Mycobacterium tuberculosis* drug resistance

**DOI:** 10.1038/s41467-026-72431-7

**Published:** 2026-04-24

**Authors:** Vishant Boradia, Junxi Chen, Andrew Frando, Lindsay V. Clark, Christoph Grundner

**Affiliations:** 1 Center for Global Infectious Disease Research, Seattle Children’s Research Institute, Seattle, WA USA; 2 Bioinformatics and Research Scientific Computing, Seattle Children’s Research Institute, Seattle, WA USA; 3 Department of Pediatrics, University of Washington, Seattle, WA USA; 4 Department of Global Health, University of Washington, Seattle, WA USA; 5 Present Address: Department of Medicine, University of Washington, Seattle, WA USA

**Keywords:** Antibiotics, Tuberculosis

## Abstract

The mycobacterial outer membrane (OM) creates a formidable permeability barrier, and whether drugs traverse it by mechanisms other than passive diffusion remains unclear. The proline-glutamic acid (PE) and proline-proline-glutamic acid (PPE) proteins of pathogenic mycobacteria include several OM transporters. Because bacterial transporters are also major contributors to drug resistance, we tested the role of PE/PPE proteins in *Mycobacterium tuberculosis* (*Mtb*) drug susceptibility. We identified mutations in multiple *pe/ppe* genes that were strongly associated with drug resistance in a genetic association study. A mutation in *ppe42* linked to clinical amikacin resistance also conferred higher amikacin resistance in vitro. Deletion of *ppe51* led to in vitro resistance to multiple drugs and was accompanied by upregulation of inner membrane efflux pumps. Deletion of a *pe/ppe* pair that responded transcriptionally to drug exposure, *pe25/ppe41*, led to increased resistance to isoniazid (INH) in strains across all major *Mtb* lineages and accelerated the emergence of INH resistance in vitro. These data show a role of several *Mtb* PE/PPE proteins in drug resistance consistent with the PE/PPE transporter paradigm and suggest a wider role of the PE/PPE family in *Mtb* drug susceptibility and clinical drug resistance.

## Abstract (web summary)

Mycobacterium tuberculosis has a complex outer membrane that often blocks antibiotics from entering the cell. A special class of proteins called PE/PEE, contains transporters that might aid in drug susceptibility. Here, they identify key mutations in these proteins that correspond to increased antibiotic resistance.

## Introduction

The outer membrane (OM) of mycobacteria consists of long-chain fatty acids that severely restrict solute diffusion^1^. This OM barrier is also highly impermeable to many clinical drugs and at least partially responsible for the high intrinsic drug tolerance of *Mycobacterium tuberculosis* (*Mtb*) and other pathogenic mycobacteria^2^. While non-pathogenic mycobacteria and gram-negative bacteria with a comparable OM express porins to facilitate free diffusion, *Mtb* and other pathogenic mycobacteria lack canonical porins. The absence of porins likely contributes to lower OM permeability of mycobacteria, which is typically two to three orders of magnitude lower than that of gram-negative bacteria^3^ and poses one of the many challenges to effective tuberculosis (TB) treatment.

A unique feature of the genomes of pathogenic mycobacteria is the presence of two large gene families with mostly unknown function, the *pe* and *ppe* genes. *Mtb*, for example, encodes approximately 100 PE and 69 PPE proteins, which together take up nearly 10% of the genome’s coding capacity^4^. Despite their large number, a unifying or shared biochemical function for the PE/PPE proteins has not yet been discovered, but diverse roles in generally shaping the host-pathogen interface have been described, consistent with their presumed localization in the OM. Several recent studies now show that some PE/PPE proteins function as specific pores or channels in nutrient transport across the OM^5–8^, and pore formation has now directly been shown for one PPE protein^9^. This emerging transport function aligns with the relative occurrence of PE/PPE proteins and porins in mycobacteria: PE/PPE proteins are typically present in much larger numbers when porins are absent^8^.

TB is exceedingly difficult to treat due to several host and bacterial factors. In addition to its impermeable OM, *Mtb* expresses inner membrane efflux pumps that effectively reduce intracellular drug concentrations^10^. Efflux pumps are commonly upregulated in clinical drug-resistant strains and are a major contributor to *Mtb* drug tolerance and resistance to virtually all drugs currently in use^11^. In contrast to inner membrane transport, how drugs traverse the impermeable OM of pathogenic mycobacteria in either direction in the absence of porins remains an open question.

Here, we show that several PE/PPE proteins affect drug resistance in a way that is consistent with transporter function and are likely facilitating drug uptake across the OM. These data challenge the idea that new TB drugs need to be lipophilic to penetrate the OM and show that the *pe/ppe* genes contribute to clinical drug resistance. With 169 *pe/ppe* genes in *Mtb*, these gene families could widely affect drug susceptibility and be a central contributor to TB drug resistance.

## Results

### Mutations in *pe/ppe* genes are associated with clinical drug resistance

**Fig. 1:**
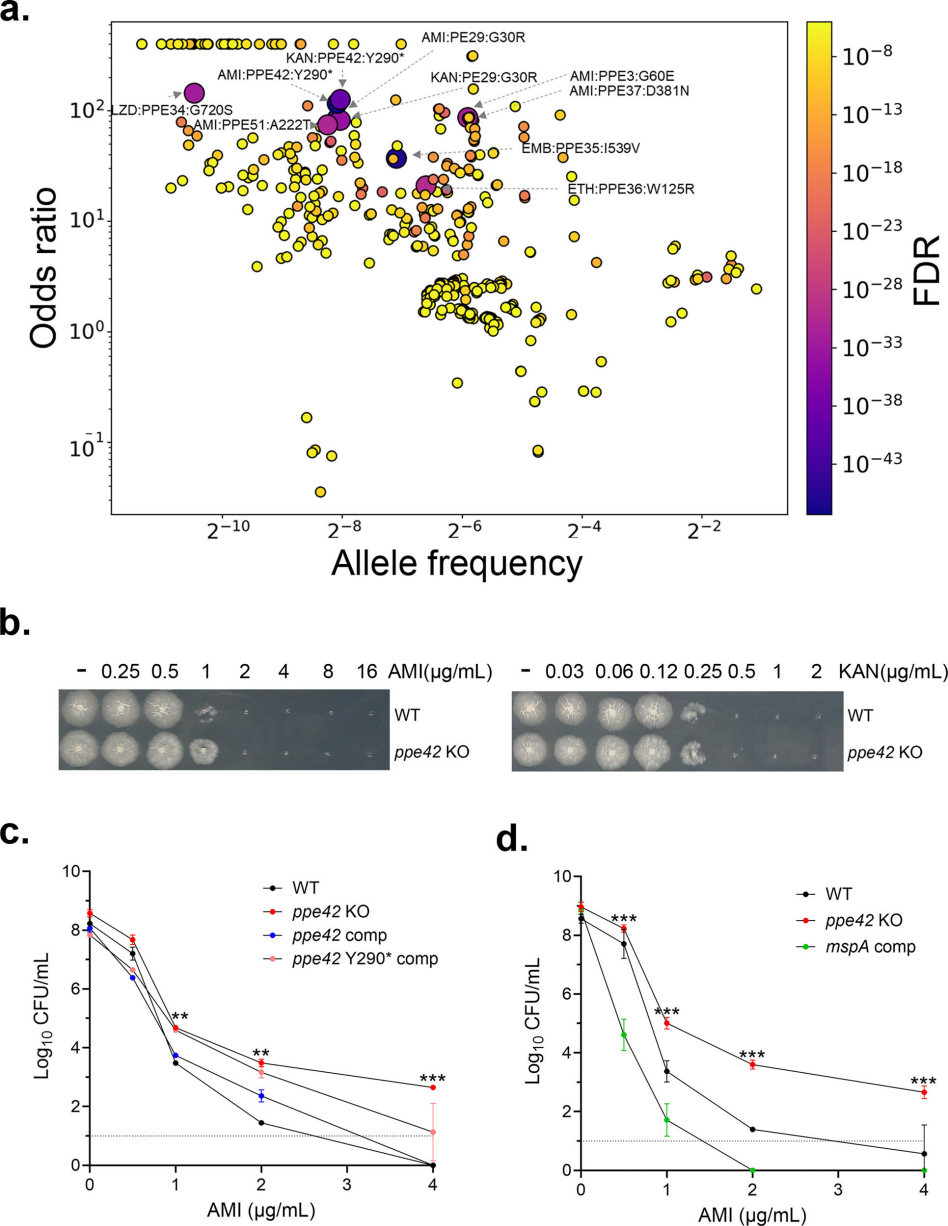
Multiple *pe/ppe* mutations are associated with clinical drug resistance. **a** Genetic associations of *pe/ppe* mutations and clinical drug resistance are shown as scatterplot with odds ratio and allele frequency. False discovery rate (FDR) is shown according to color scheme (right). Larger symbols indicate the top ten candidates based on FDR, and the respective drug and mutation are indicated. *pe-pgrs* genes were excluded from the analysis. **b** Spotting assay after exposure to drug shows that deletion of *ppe42* increases resistance to amikacin, but not kanamycin. Cultures were grown with drug for 7 days before spotting on agar without drug. **c** Deletion of *ppe42* is partially complemented by WT *ppe42* but not the Tyr290 truncation, as shown by CFU assay. **d** The MspA porin reverses the resistance of the *ppe42* KO to amikacin. Error bars for CFU assays show mean ± SD of three biological replicates. Significance was calculated using two-sided multiple comparison *t-*test and *P*-values for comparisons in (**c**) and (**d**) are given in Supplementary Data 2. Dotted line in (**c**) and (**d**) denotes limit of detection. Abbreviations: AMI amikacin, EMB ethambutol, KAN kanamycin, LZD linezolid. We used Gemini and ChatGPT for writing a Python script to generate Fig. 1a. We reviewed and validated all AI outputs before inclusion.

To identify candidate *pe/ppe* genes that may contribute to clinical drug resistance, we carried out a targeted genetic association study with a collection of 16,891 genome sequences of drug- and multi-drug resistant *Mtb*. Whole genome sequence data of *Mtb* was obtained from three sources: 2659 genome sequences from the Wellcome Trust Sanger Institute pilot study^12^, 3922 from the NIH-NIAID TB Portals collection^13^, and 10,310 from the CRyPTIC study^14^. We analyzed sequences of the *pe* and *ppe* genes along with known resistance loci (*aphC, gyrA, inhA, katG*, and *rpoB*) as positive controls. Because of their high sequence variability and complex repetitive sequences, DNA sequence quality and genome assembly across the *pe*/*ppe* genes can be poor when using short-read sequencing, which is predominantly used for sequencing of drug resistant *Mtb*. As a result, many GWAS studies excluded *pe*/*ppe* genes from analysis. To assess data quality and possible bias, we analyzed the read depth and mapping quality across *pe*, *ppe*, and *pe-pgrs* genes from the set of nearly 4000 genomes from the NIH collection. Read depth was generally comparable between *pe/ppe* and all other genes. Mapping quality for the *pe* genes was also comparable to all non-*pe*/*ppe* genes but was reduced for the *ppe* genes and more so for the *pe-pgrs* genes (Supplementary Fig. 1). The *pe-pgrs* subfamily is also known to harbor large sequence variation independent of drug resistance^15–17^ and together with the *ppe-mptr* subfamily is the main source of errors in *Mtb* genome sequences^18^. This variation and the reduced mapping quality led us to exclude the *pe-pgrs* genes from further analysis. We considered promoter and gene body mutations for the genetic association analysis. Using the linear mixed model approach in PySEER, some of the most significant associations were found with mutations in *ppe42*, *ppe35*, and *ppe51* (Fig. 1a). The *ppe42* mutation most significantly associated with drug resistance introduces a premature stop codon and was previously noted by the CRyPTIC consortium to be associated with amikacin and kanamycin resistance^14^. Mutations in *ppe35* have also previously been linked to pyrazinamide resistance^19,20^, and *ppe51* has experimentally been implicated in resistance to isoniazid (INH), although a transporter or channel role for PE/PPE proteins was not recognized at the time^21^. Additional associations with drug resistance that had high odds ratios and low false discovery rate (FDR) were found in *ppe3*, *pe29*, and *ppe34-37* (Fig. 1a, Table 1). Further filtering to associations with FDR < 0.00001, we identified 35 promoter and 443 coding variants associated with resistance to at least one drug within our target genes, in addition to the five positive controls (Supplementary Data 1).

**Table 1:** *pe* and *ppe* genes associated with drug resistance in the genetic association study. The ten mutations with the lowest FDR and odds ratio >10 are shown

Gene	Drug	Variant	FDR global	*P*-value	Allele frequency	Odds ratio
*ppe42*	Amikacin	Y290*	4.86E-48	1.94E-68	3.69E-03	116.36
*ppe35*	Ethambutol	I539V	1.35E-46	1.30E-56	7.31E-03	37.07
*pe29*	Amikacin	G30R	1.02E-41	2.88E-64	3.69E-03	84.67
*ppe42*	Kanamycin	Y290*	8.74E-40	5.45E-52	3.82E-03	126.06
*pe29*	Kanamycin	G30R	9.02E-35	5.84E-49	3.82E-03	81.76
*ppe3*	Amikacin	G60E	8.64E-33	6.79E-280	1.67E-02	86.47
*ppe51*	Amikacin	A222T	1.10E-31	5.87E-56	3.30E-03	74.39
*ppe36*	Ethionamide	W125R	8.77E-31	1.50E-62	1.03E-02	20.87
*ppe37*	Amikacin	D381N	1.65E-30	6.21E-278	1.68E-02	82.34
*ppe47*	Amikacin	E124G	2.63E-30	8.90E-278	1.66E-02	85.79

### *ppe42* mutation leads to amikacin resistance

To test whether the *ppe42* mutation is causal for amikacin and/or kanamycin resistance, we generated a *ppe42* deletion strain by recombineering^22^, and complemented this strain with wild-type (WT) and *ppe42* with the associated clinical mutation, a stop codon at Tyr290. The complex OM lipids Phthiocerol Dimycocerosates (PDIM) can be lost during in vitro culture and affect OM permeability^23,24^. To maintain PDIM, we grew all cultures in 100 µM sodium propionate^24^ and tested for intact WT sequence of the PDIM biosynthetic gene cluster by RNA-seq (Supplementary Table 1). We did not detect any mutations and only minor differences in RNA abundance for PDIM biosynthesis genes in the WT or deletion strains used here. Because the association of a genetic mutation especially with second-line drugs is often confounded by multiple drug-resistance, we tested against a panel of ten drugs currently in clinical use. We incubated liquid culture of H37Rv WT and the *ppe42* deletion mutant in the presence of drugs for seven days and determined cell viability by spotting on solid medium. The *ppe42* deletion strain was more resistant to amikacin, a second-line drug used for treatment of multi-drug resistant TB^20^, but not kanamycin or any other drug tested when compared to WT (Fig. 1b, Supplementary Figs. 2, 3). We next plated amikacin-treated strains on solid media for quantitating the difference in amikacin susceptibility by colony forming units (CFU) assay. At amikacin concentrations near the minimal inhibitory concentration (MIC), the *ppe42* deletion mutant was more resistant, with ~2 log higher CFU than the WT. The *ppe42* deletion strain regained some sensitivity to amikacin when complemented with WT *ppe42*, but not when complemented with the copy of *ppe42* that carried the clinical mutation (Fig. 1c, see Supplementary Data 2 for all *P*-values, CFU data for kanamycin in Supplementary Fig. 2). Since the mutated *ppe42* phenocopies the deletion strain, the clinical mutation is likely a loss of function mutation. To test if the increased drug resistance of the *ppe42* deletion strain is related to transport across the OM, we next tested if the *M. smegmatis* porin MspA can functionally complement the *ppe42* deletion. MspA has previously been shown to be an OM porin in nonpathogenic mycobacteria that can bypass the OM when introduced into pathogenic mycobacteria^25^. To test if this OM porin can revert the phenotype of *ppe42* deletion, we expressed *mspA* in the *ppe42* knockout strain. Expression of *mspA* fully restored amikacin susceptibility in the *ppe42* deletion background (Fig. 1d). We next analyzed the *ppe42* deletion strain by RNA-seq to test for secondary effects of the deletion on other genes, for example efflux pumps. The *ppe42* deletion strain showed modest upregulation of Rv2609c and *pdxH*, two genes directly adjacent to *ppe42* (Supplementary Fig. 4, all differentially expressed genes in Supplementary Data 3). This effect is likely a gene neighborhood effect that is not relevant for the *ppe42* deletion phenotype since the deletion strain could be complemented with a WT copy of *ppe42*. These data show that the truncation at Tyr290 in *ppe42* observed in clinical drug resistance confers drug resistance to amikacin but not kanamycin.

### *ppe51* deletion leads to resistance to multiple drugs

**Fig. 2:**
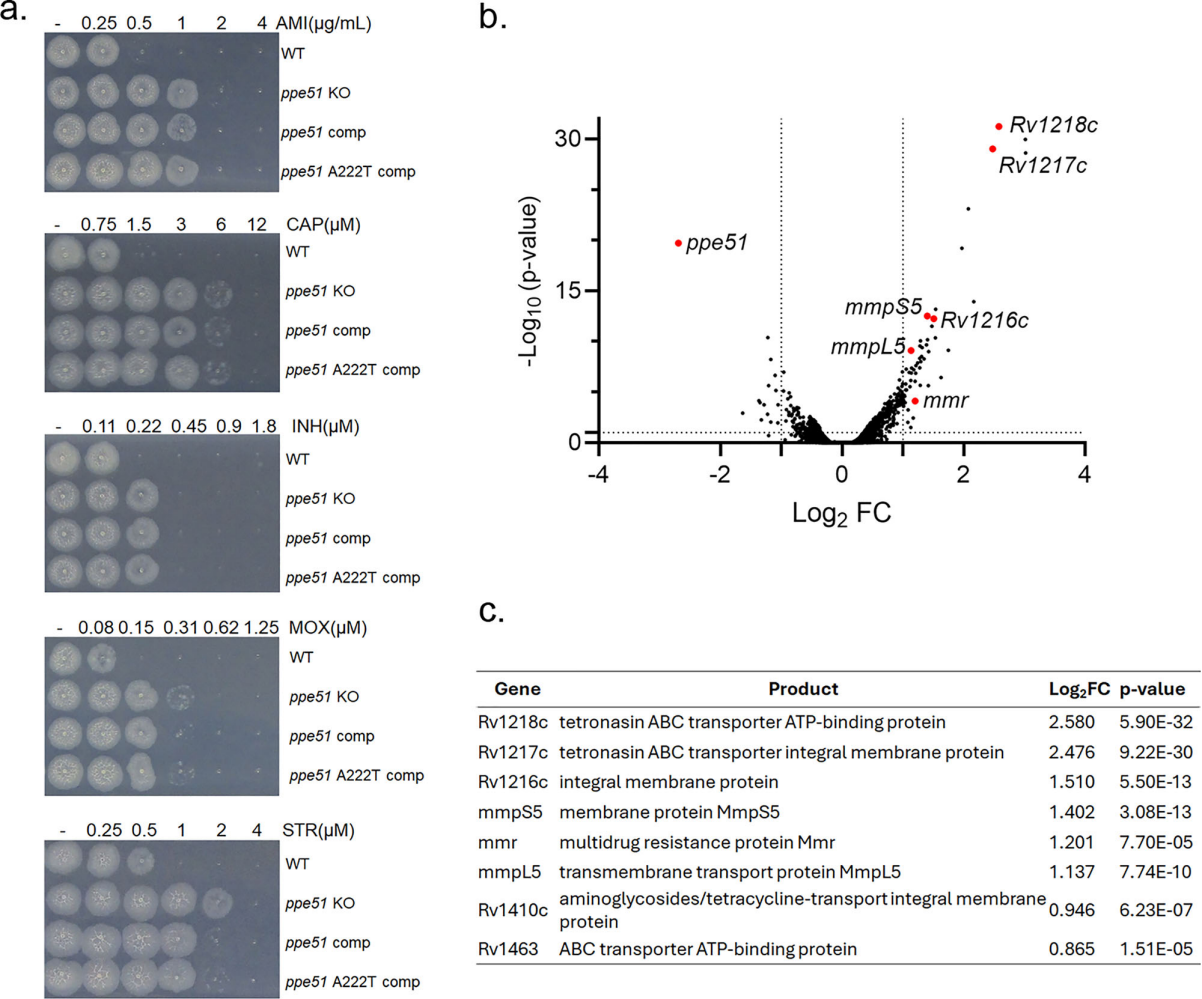
Deletion of *ppe51* increases drug resistance and upregulates inner membrane transporters. **a** Spotting of WT and *ppe51* KO strain grown in presence of drugs in 2-fold dilution shows increased resistance to multiple drugs. **b** RNA-seq analysis shows increased transcription of several known efflux pump genes as a result of *ppe51* deletion. **c** The efflux pump genes affected by *ppe51* deletion. All differentially expressed genes are given in Supplementary Data 2. Gene expression changes were identified using a combination of five differential expression tools within DuffyTools: round robin, RankProduct, significance analysis of microarrays (SAMs), EdgeR, and DeSeq2. The results from each DE tool were combined using a weighted average of fold change and significance (*P-value*). Abbreviations: AMI amikacin, CAP capreomycin, STR streptomycin, MOX moxifloxacin, INH isoniazid.

To test for a role of *ppe51* in drug resistance, we generated a *ppe51* deletion strain and tested its susceptibility to drugs. The *ppe51* deletion strain showed increased resistance to amikacin, capreomycin, moxifloxacin, streptomycin, and isoniazid (Fig. 2a). Because deletion of *ppe51* conferred resistance to multiple drugs, we explored potential compensatory genetic effects of *ppe51* deletion by comparing global transcription in the WT and deletion strains by RNA-seq. Deletion of *ppe51* led to multiple significant changes in gene expression compared to WT (Fig. 2b). Notably, transcripts for six inner membrane efflux pump genes (*mmr*, *mmpL5*, *mmpS5*, *Rv1216c-1218c*) were significantly induced (Fig. 2b, c, all differentially expressed genes in Supplementary Data 3). Since these genes are known to efflux at least INH^11^, the effect of *ppe51* deletion on drug resistance may be indirect through upregulation of inner membrane efflux pumps and indicates coupling of *ppe51* with inner membrane transport. We next tested drug susceptibility of the *ppe51* deletion strain complemented with a WT *ppe51* gene and a *ppe51* gene carrying the clinical Ala222Thr mutation identified in our association study. WT partially complemented the deletion for two out of the five drugs. The Ala222Thr complement phenocopied the *ppe51* deletion strain, indicating that it is a loss-of-function mutation and responsible for drug resistance (Fig. 2a).

### *ppe3*, *ppe35*, and *ppe36* deletion do not affect drug resistance

We tested additional candidates from our association study for effects on drug resistance. We tested a *ppe35* deletion strain for resistance to pyrazinamide or any other of ten clinical drugs, but the deletion strain did not alter the sensitivity to these drugs. Similarly, deletion of *ppe3* and *ppe36* did not affect drug resistance to any of the ten tested drugs (Supplementary Fig. 5). Because *ppe35* has been noted in several studies to be associated with pyrazinamide resistance^19,20^ and the spotting assay has relatively low resolution, we tested the *ppe35* deletion mutant also by CFU assay. CFU data confirmed that deletion does not affect growth in pyrazinamide (Supplementary Fig. 6). These data show the limitations of genetic association studies, which might be particularly challenging for the *pe-pgrs* and *ppe* genes due to the poorer mapping quality of sequencing data. These data also show that deletion of *ppe* genes does not generally affect the OM permeability but only does so in the case of specific *ppe* genes and in the context of specific drugs.

### *pe25/ppe41* affect susceptibility to INH

**Fig. 3:**
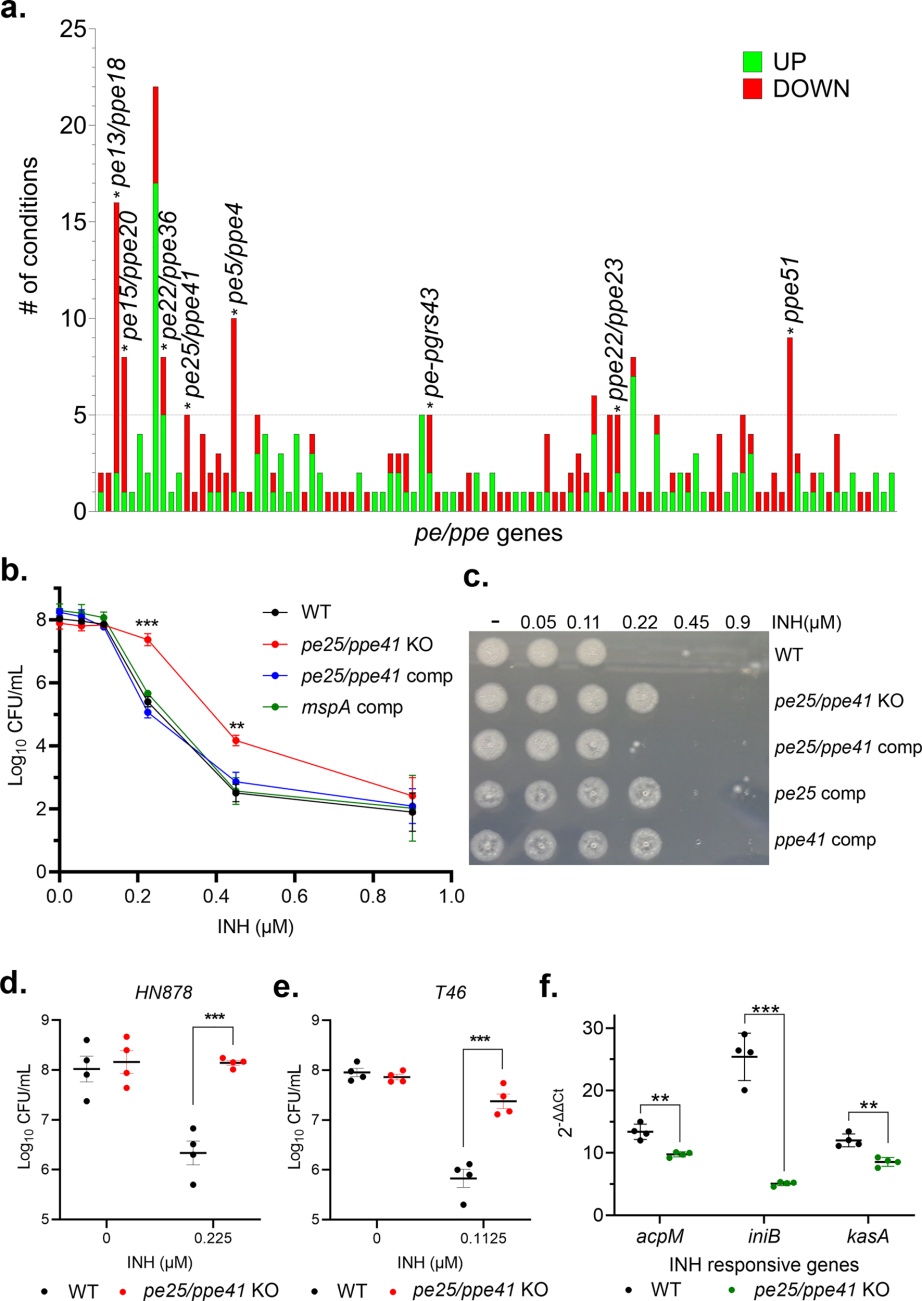
The PE25/PPE41 complex affects INH susceptibility. **a** The transcriptional response of *pe/ppe* genes to drugs. *pe/ppe* genes showing transcriptional up- (green) or downregulation (red) in response to drugs in historical RNA-seq data were plotted against the number of drugs they were found to respond to. Deletions of *pe* and *ppe* genes tested in this study are labeled. Direction of change is indicated by color. **b** A *pe25/ppe41* deletion strain shows higher resistance to INH. The heterologous OM porin MspA from *M. smegmatis* can functionally complement the *pe25/ppe41* deletion. Bacteria were enumerated by colony forming units (CFU) assay and data are shown as the mean ± SD of three biological replicates. Statistical significance was determined by two-sided multiple comparison *t*-test. **: *P* < 0.01, ***: *P* < 0.001. **c** Complementation of the *pe25/ppe41* deletion restores susceptibility to INH, but not complementation with either *pe25* or *ppe41* alone, as shown by spotting assay. **d** Deletion of *pe25/ppe41* also reduces the susceptibility to clinically relevant concentrations of INH in *Mtb* strains from lineage 1 (T46) and **e** lineage 2 (HN878). Bacteria were enumerated by colony forming units (CFU) assay and datapoints show the average of two technical replicates for four biological replicates. Error bars are mean ± SEM. Significance was calculated using unpaired *t*-test **f** The known INH-responsive genes *iniB*, *acpM*, and *kasA* are induced by INH exposure in the H37Rv WT but less so in the *pe25/ppe41* deletion strain, as determined by qRT-PCR. Datapoints show the average of two technical replicates for four biological replicates **: *P* < 0.01, ***: *P* < 0.001. Error bars are mean ± SD. Significance was calculated using unpaired t-test. All the *P*-values are mentioned in supplementary data 2. Abbreviations: INH: isoniazid.

We next sought to identify additional candidates by an orthogonal approach. Transporters can respond transcriptionally to their substrates, a behavior that has also been reported for several *pe/ppe* transporters^6,8^. To identify additional *pe/ppe* genes that might affect drug resistance but were not detected by our genetic association study, we mined publicly available RNA-seq data for changes in *pe/ppe* gene expression that is induced by drugs. Twenty-eight datasets from public repositories captured the transcriptional responses to ten drugs. From these data, we identified the *pe/ppe* genes that were significantly regulated in response to drug exposure and plotted them against the number of drugs to which they responded (Fig. 3a). Many *pe/ppe* transcripts responded to drug exposure, and several to different drugs. Generally, more *pe/ppe* transcripts were down- than upregulated in response to drugs (Fig. 3a). To test if these candidates have any functional consequence for drug resistance, we generated deletion strains for five and tested their susceptibility with the ten-drug panel by spotting assay. The *pe25/ppe41* deletion strain was more resistant to INH than WT. To quantitate the effect of *pe25/ppe41* deletion on drug resistance, we determined the MIC of the WT and deletion strains by CFU assay. At concentrations of INH corresponding to the MIC of drug-susceptible *Mtb* (0.225-0.45 µM), the *pe25/ppe41* deletion strain was more resistant than the WT strain, with >10-fold higher CFU and ~2-fold increase in the MIC_50_ (Fig. 3b). Complementation of the *pe25/ppe41* deletion strain fully restored the susceptibility to INH (Fig. 3b). None of the other deletion strains we tested showed altered drug susceptibility (Supplementary Fig. 7). As before, all strains were grown in propionate to maintain PDIM, and strains were tested by RNA-seq for integrity of PDIM biosynthesis genes.

To test if the increased drug resistance of the *pe25/ppe41* deletion strain is related to transport across the OM, we next tested if the *M. smegmatis* porin MspA can functionally complement the *pe25/ppe41* deletion. MspA restored the susceptibility of the *pe25/ppe41* deletion strain to INH to WT levels, suggesting that PE25/PPE41 has a similar function to MspA in transport across the OM (Fig. 3b). The decreased susceptibility to INH upon *pe25/ppe41* deletion indicates that the PE25/PPE41 complex imports INH. To test if *pe25*, *ppe41*, or both affect INH sensitivity, we complemented the *pe25/ppe41* deletion strain with each gene individually under the control of an anhydrotetracycline (ATc)-inducible promoter. Complementation with both but not with *pe25* or *ppe41* alone restored sensitivity to INH, showing that both are required (Fig. 3c). The genetic background of a strain can affect drug resistance phenotypes^26,27^. To test the effect of strain background and whether the *pe25/ppe41* effect on INH susceptibility is conserved beyond the lineage 4 strain H37Rv, we introduced *pe25/ppe41* deletions in representative strains from the major circulating lineages—T46 (lineage 1) and HN878 (lineage 2). Both strains showed similar changes in INH resistance to the lineage 4 reference strain H37Rv (Fig. 3d, e). To test for any genetic effects of *pe25/ppe41* deletion, we analyzed RNA-seq data of WT and the deletion strain (Supplementary Fig. 8). The deletion had only minor effects on transcription, with the largest changes in *ahpD*, a gene upstream of *ppe41*, suggesting gene neighborhood effects unlikely to affect the deletion phenotype because they could also be reverted by complementation with WT *pe25/ppe41*.

### Loss of *pe25/ppe41* reduces transcription of INH-responsive genes

INH is a prodrug that requires activation by KatG in the cytoplasm, and it has well-defined transcriptional effects^28^. To further test for an INH import function of *pe25/ppe41* and to test whether *pe25/ppe41* alone can alter the levels of INH in the cytoplasm, we tested if *pe25/ppe41* deletion affects the expression of INH-responsive genes. We chose the three INH-responsive genes *iniB*, *acpM*, and *kasA*, and compared their expression by qRT-PCR in WT and the *pe25/ppe41* deletion strain after treatment with 0.2 µg/mL INH for 5 hours. INH strongly induced the expression of the three INH-responsive genes in WT but significantly less in the *pe25/ppe41* deletion strain (Fig. 3f). These data indicate that *pe25/ppe41* deletion is sufficient to reduce INH concentrations in the cytoplasm, which is consistent with an importer function.

### *pe25/ppe41* mutations accelerate drug resistance

**Fig. 4:**
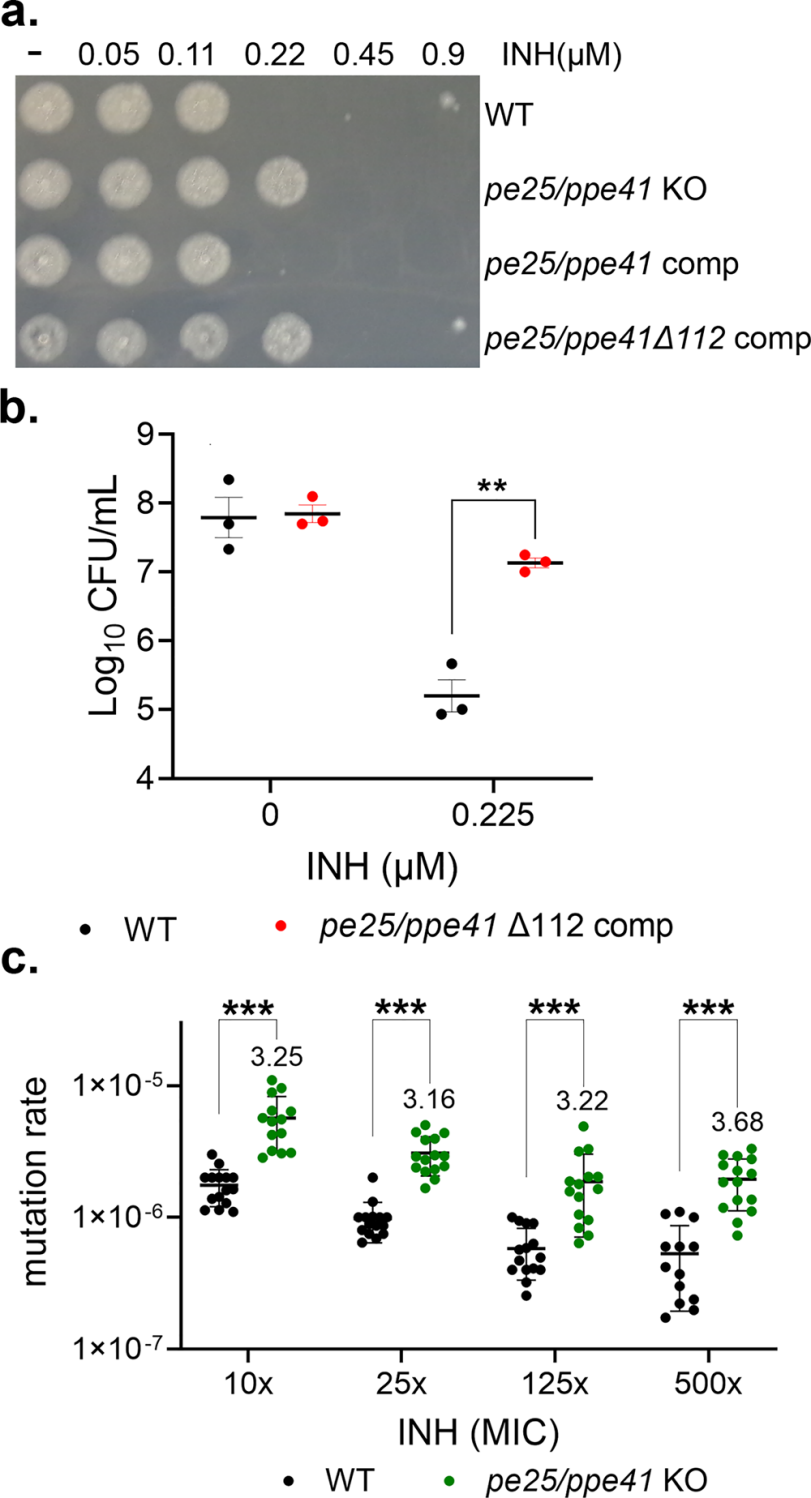
*ppe41* truncation contributes to INH resistance. **a** The *ppe41* deletion at bp112 in the Lisboa3 strain family increases resistance of *Mtb* to INH, as shown by spotting after growth on drug for 7 days. **b** *ppe41* truncation increases INH resistance as quantified by CFU assay. Data points represent average of two technical replicates for three biological replicates. Error bars show mean ± SEM. Significance was calculated using unpaired *t*-test. **c** Deletion of *pe25/ppe41* increases the rate at which INH resistance emerges. *Mtb* cultures were plated onto 7H10 + GO plates without drug and with INH at concentrations 10x–500x above the MIC. Colonies on drug-free plates represented the total viable cell count, while colonies on plates with drug were counted as resistant mutants. The mutation rate was calculated by dividing the number of colonies on drug-containing plates by the total viable cell count from non-drug plates plates. Each datapoint represents a biological replicate. Error bars are mean ± SD. Statistical significance was determined by two-sided multiple comparison t-test. ***: *P* < 0.001. All *P*-values are mentioned in supplementary data 2. Abbreviations: INH: isoniazid.

Although neither *pe25* nor *ppe41* mutations were significantly associated with drug resistance in our genetic association analysis, anecdotal data also link *pe25/ppe41* to clinical drug resistance: The Lisboa cluster of *Mtb* strains has been associated with INH resistance and high rates of multidrug-resistant and extensively drug-resistant TB in Portugal^29–31^. Although INH resistance in these strains is associated with two canonical *inhA* mutations^32^, strains in the Lisboa3 cluster also carry a truncation of *ppe41* after bp112^30^. To test if this *ppe41* truncation could also contribute to INH resistance, we complemented the H37Rv *pe25/ppe41* deletion strain with a WT or truncated copy of *ppe41*. In contrast to the WT gene, the truncated *ppe41* did not restore INH sensitivity of the *pe25/ppe41* deletion strain (Fig. 4a), suggesting that it contributes to high-level INH resistance in the Lisboa3 strain and/or promotes the emergence of canonical INH mutations. To quantitate the effect of the truncation, we determined the CFU of the WT strain and a *pe25/ppe41* deletion strain complemented with the truncated *ppe41*. The truncated *ppe41* conferred higher resistance, with ~2 orders of magnitude higher CFUs (Fig. 4b). To test the idea that *pe25/ppe41* deletion can affect the rate of emergence of INH resistance, we plated WT and the *pe25/ppe41* deletion strain on agar plates containing INH at 10-, 25-, 125-, and 500-fold the MIC and quantified the resistant colonies that grew under drug pressure. The *pe25/ppe41* deletion strain developed INH-resistant colonies at an about 3.5-fold higher rate than the WT strain across all tested concentrations (Fig. 4c). Sequencing of resistance mutants showed that resistance in the *pe25/ppe41* deletion strain was due to canonical *katG* mutations typically associated with INH resistance in vitro (Supplementary Fig. 9). These findings indicate that loss of *pe25/ppe41* function can accelerate the emergence of INH resistance.

## Discussion

*Mtb* and related pathogenic mycobacteria have some of the thickest and most impermeable OMs known in microbiology^1,33^. The mechanism by which *Mtb* takes up nutrients through this thick barrier in the absence of canonical porins was a longstanding question until the first *Mtb* transporters from the PE/PPE protein families were discovered. The PE/PPE proteins are an idiosyncratic protein family with no apparent orthologs outside of mycobacteria, and they are generally more frequent in pathogenic mycobacteria. With their large number, they have surfaced in numerous screens and studies, suggesting a perplexing number of functions and cellular roles, mostly in shaping interactions with the host cell. Several PE/PPE proteins have now been shown to be nutrient transporters or channels across the OM^6,8^—perhaps the most unifying explanation of PE/PPE function yet and one that is compatible with many previous observations.

Drug uptake by *Mtb* is generally presumed to occur by passive diffusion, an idea that has led to a guiding principle in TB drug development that TB drugs should be lipophilic. However, many effective TB drugs in clinical use are not. When interpreted in the context of transporter function, the effects of PPE42 on amikacin susceptibility, of PPE51 potentially on several drugs, and of PE25/PPE41 on INH susceptibility would be consistent with an importer function, and the presence of proteinaceous OM drug importers would provide a plausible explanation for this contradiction. With the exception of PPE51, the effects of the PE/PPE proteins tested here on drug susceptibility are highly specific. Of nine *pe/ppe* tested, only three showed any altered drug susceptibility, two only to one drug. These data suggest that *pe/ppe* deletion does not cause a generalized permeability change in the OM but has specific effects on specific compounds. The same may not be true for the *pe/ppe* deletion strains complemented by *mspA*: Although *mspA* in all cases functionally complemented *pe/ppe* deletion, consistent with providing an alternative path through the OM, other, more general effects of MspA on OM integrity that could also contribute to complementation of the *pe/ppe* deletion phenotypes have been reported, at least in *M. smegmatis*^34,35^.

The function of PPE51 appears to be more complicated. Not only do we show coupling of the *ppe51* gene to expression of some inner membrane efflux pumps; consistent with our data, previous work also showed an effect of *ppe50/ppe51* deletion on INH and RIF resistance^21^, and the *ppe51* paralogs in *M. marinum* affect general OM permeability^36^, although with the opposite effect on drug resistance as in *Mtb*. *ppe51* loss-of-function mutations are under positive selection in multidrug-resistant *Mtb*, suggesting a potential role in compensating for primary resistance mutations^37^. Complementation of the ppe51 deletion strain with WT and the Ala222Thr mutation were not fully consistent: Although the lack of complementation with the Ala222Thr mutant with any of the five drugs could suggest that it is causal for clinical drug resistance, the WT gene only complemented with two out of the five drugs, and not to WT levels in any of the experiments. Without full complementation with the WT *ppe51* gene, the effect of the Ala222Thr mutation in these complementation experiments remains ultimately inconclusive. These data also indicate more complicated effects of *ppe51* that are not readily reversed by complementation. Although we consider increased efflux as a plausible explanation for the drug phenotypes seen here, these other effects might also contribute to its function.

In addition to mutations in *ppe42* and *ppe51*, loss of function mutations of *pe25/ppe41* could be a facile mechanism for drug tolerance and/or resistance. Our data indeed show a higher rate of emergence of INH resistance in the *pe25/ppe41* deletion strain in vitro, suggesting it could be a steppingstone mutation to high-level drug resistance, for example in some Lisboa strains. The contribution of *pe/ppe* genes to clinical drug resistance combined with the large number of PE/PPE proteins in *Mtb* represent a previously unrecognized factor of TB drug resistance and raise the possibility that PE/PPE proteins contribute to drug resistance more widely. Several observations indeed point to a larger role of PE/PPE proteins in drug susceptibility and resistance. A genome-wide chemical-genetic screen recently identified several *pe/ppe* genes that conferred increased or decreased susceptibility to drugs in vitro^38^. These data are consistent with our findings for *pe25/ppe41* and INH but require experimental validation for others since CRISPRi can cause polar effects, and expression of *pe/ppe* genes can be linked to expression of inner membrane transporters—as we have shown for *ppe51*. Also, the large number of *pe/ppe* genes makes redundancy likely, potentially masking the effects of single gene deletion or knockdown and complicating the identification of PE/PPEs that affect drug susceptibility and resistance.

The physiologic substrate(s) and cellular function(s) of PPE42 and PE25/PPE41 remain to be identified. Although drugs are arguably accidental substrates of PE/PPE transporters, their cellular substrates might bear chemical resemblance to the drugs they transport. Interestingly, PPE42 contains a predicted serine hydrolase domain. Serine hydrolases play an important role in the resistance to beta-lactams (such as amikacin and kanamycin) through drug inactivation. The predicted AlphaFold structure for the PPE42 hydrolase domain shows a canonical catalytic site organization and a Ser residue in the expected position, suggesting it might be an active enzyme. However, loss of the hydrolase domain in the *ppe42* clinical resistance strain would have the opposite effect to known hydrolase-mediated drug resistance mechanisms and not lead to drug inactivation. The molecular function of the hydrolase domain in amikacin resistance needs further study, but it could serve as a binding domain for drug and thus facilitate entry. Our data identify a function for PE/PPE proteins in drug susceptibility that is consistent with their emerging OM transporter, or channel function. Although transporter function of PE/PPE proteins has now been shown in several cases, and many previously observed PE/PPE phenotypes are fully consistent with transporter function^7,39^, much remains unknown about these unique protein families. For example, the topology and composition of the complexes, which are and which are not pore-like, the way in which pores or channels are formed across the OM, and the biochemical mechanism of transport all remain unknown but will inform drug development, drug targeting, diagnosis of drug resistance, and new strategies to counter drug resistance.

## Methods

### Genetic association analysis

Sequences were aligned to the MT_H37R7_V3 reference sequence using BWA-MEM^40^, and the assembled genomes were aligned to the same reference using Minimap2^41,42^. The 17,047 files were then processed by BCFtools mpileup and BCFtools call^43^ to make a joint-called VCF with haploid genotype calls. We considered promoter and gene body mutations for the analysis. A selection of 7,402 SNPs with less than 10% missing data in all three datasets and a minor allele count of at least 5, representing a random 10% of genomic regions, were used for estimating relatedness among strains. Relatedness was estimated by multiplying the centered genotype matrix by its transpose. Individual relatedness values were divided by the number of non-missing variants in common between two samples, and negative relatedness values were set to zero. The lineage of each strain was identified based on the set of lineage-specific SNPs packaged with TBprofiler v6.5.0^44^. Samples were retained if they had less than 10% missing data, showed agreement between assigned lineage and relatedness to other strains, and had antibiotic resistance phenotype data available, leaving 16,891 strains. We tested the 104 *pe/ppe* genes for association with phenotypic drug resistance. We excluded the *pe-pgrs* subfamily (64 genes) because it is known to have highly variable sequence. We included *aphC*, *gyrA*, *inhA*, *katG*, and *rpoB* as controls with known resistance associations. Within these 109 genes, we identified 19,154 nucleotide variants after splitting multiallelic variants into individual biallelic variants. Association analysis was performed at the nucleotide level for promoter variants and amino acid level for coding variants. Coding variants were agglomerated by amino acid position and type of variant (substitution, frameshift, in-frame insertion or deletion). Any given promoter or coding variant was used in association analysis with a given drug resistance trait if it had at least five strains with the minor allele and a recorded phenotype, totaling 357 unique promoter variants and 3418 unique agglomerated coding variants across all 25 drugs. Association testing was conducted with PySeer^45^ using the linear mixed model approach, with the relatedness matrix as a random effect (similarity matrix), and dataset (Sanger Institute, CRyPTIC, TB Portals) as a fixed covariate. At FDR < 0.00001 across all tests, 35 promoter variants and 478 coding variants were significant for resistance to at least one drug. If the number of susceptible individuals with the minor allele was required to be zero, and the minor allele had to appear in resistant strains of at least two major *Mtb* lineages, there were six promoter variants and 41 coding variants significant for resistance to at least one drug within our target genes, including the five positive controls and 11 out of the 104 *pe/ppe* genes.

### Media and growth conditions

*Mycobacterium tuberculosis (Mtb*) H37Rv, a lineage 4 strain, was used as the parental strain for generating all mutants unless indicated otherwise. Strains were grown in Middlebrook 7H9 medium (Difco), supplemented with 10% (vol/vol) oleic acid-albumin-dextrose-catalase (OADC) enrichment (BBL; Becton Dickinson), 0.5% glycerol, and 0.05% Tween 80 or 0.05% Tyloxapol. The medium is referred to as “7H9 + GO” when supplemented with glycerol and OADC only, “7H9+GOTy” with Tyloxapol, and “7H9+GOTyP” with the addition of 100 µM sodium propionate to maintain PDIM levels^24^. For solid media, 7H10 agar was supplemented with 10% OADC and 0.5% glycerol. Strains harboring antibiotic resistance cassettes were cultured with the appropriate antibiotic: 50 μg/mL hygromycin, 30 μg/mL kanamycin, or 25 μg/mL zeocin. All drugs used for the drug screening assay were purchased from Sigma, and stock solutions were prepared in either water or DMSO.

### Creation of deletion and complemented strains

The deletion strains were generated using recombineering, as described previously^22^. Initially, 500 bp upstream and downstream of the gene of interest, along with the hygromycin resistance cassette (primers Hyg_F and Hyg_R), were amplified separately (all primers used for each deletion are listed in Supplementary Data 4). The PCR fragments were then Gibson ligated to the 5′ and 3′ ends of the hygromycin resistance cassette to create the recombineering cassette. This linear recombineering cassette was PCR-amplified, purified, and electroporated into the *Mtb* H37Rv strain carrying the recombineering plasmid pNIT:Etc^46^. Hygromycin-resistant colonies were screened, and the positive clones were confirmed by DNA sequencing. The PE25/PPE41 deletion was also generated in *Mtb HN878* and *T46* using the same recombineering pNIT:Etc strain and cassette used for *H37Rv*.

For the complemented strains, the ppe42, ppe42 Y290*, ppe51, *ppe51 A222T*, *pe*25, *ppe41, pe25/ppe41*, and *pe25/ppe41* (Δ112) coding regions, as well as the *M. smegmatis mspA* coding region were amplified by PCR using primers listed in Supplementary Data 4 and Gibson cloned into the pDTCF plasmid (Zeocin resistance) with a C-terminal FLAG tag, under the control of an anhydrotetracycline (ATc)-inducible promoter. The resulting plasmids were electroporated into the H37Rv *pe25/ppe41* deletion strain, and positive clones were selected by growth in hygromycin and zeocin.

### Drug screening

Drug screening was carried out in 96-well plates. Cultures were grown to log phase in 7H9+GOTyP media and incubated with drugs in 2-fold dilutions at a final optical density (OD) of 0.005. For pyrazinamide, we used medium at pH6. The assay plates were incubated at 37 °C for 7 days, and cell viability was measured using either the agar spotting method, Alamar Blue assay, or colony-forming unit (CFU) counting. For the spotting method, cultures were mixed and 3 µL were spotted onto 7H10 + GO agar plates, followed by incubation at 37 °C for 2-3 weeks. For CFU counting, cultures were diluted and plated onto 7H10 agar plates, which were incubated for 3-4 weeks at 37 °C.

### RNA sequencing

H37Rv and the *pe/ppe* deletion strains were grown to an OD_600_ of 0.8 in 7H9+GOTyP medium. Cells were pelleted and resuspended in buffered water, then incubated for an additional 5 h. Following incubation, cells were pelleted at 4000 g for 5 min at 4 °C, resuspended in Trizol, and lysed by bead beating for 30 s at 6 m/s for three cycles, with intermittent cooling on ice. Cell debris was pelleted at 20,000 g for 1 min, and the supernatant was transferred to a heavy phase-lock gel tube containing 300 µL of chloroform. The tubes were inverted for 2 min and centrifuged at 20,000 g for 5 min. RNA in the aqueous phase was precipitated with 300 µL of isopropanol and 300 µL of high-salt solution (0.8 M sodium citrate, 1.2 M sodium chloride). RNA was purified using the QIAGEN RNeasy kit, and ribosomal RNA was depleted using a previously published protocol^47^. Briefly, a biotinylated oligo mixture of 23S, 16S, and 5S was incubated with RNA to anneal to rRNA, followed by incubation with streptavidin beads. mRNA was purified from the supernatant using Ampure XP beads. A cDNA library was generated using the NEBNext Ultra II RNA Library Prep Kit, and each replicate was barcoded in the DNA library using the NEBNext Multiplex Oligos for Illumina. Libraries were quantified using the KAPA qPCR quantification kit, pooled, and sequenced at the University of Washington Northwest Genomics Center using the Illumina NextSeq 500 High Output v2 Kit. The *ppe42* deletion strain and WT were sequenced at Fred Hutch Cancer Center with Illumina NovoSeqX + . Read alignment was performed using the Bowtie 2 custom processing pipeline (https://github.com/robertdouglasmorrison/DuffyNGS, https://github.com/robertdouglasmorrison/DuffyTools). Gene expression changes were identified using a combination of five differential expression tools within DuffyTools: round robin, RankProduct, significance analysis of microarrays (SAMs), EdgeR, and DeSeq2. The results from each DE tool were combined using a weighted average of fold change and significance (*p-value*). Genes with an averaged absolute fold change of more than 2-fold and a *P-value* < 0.01 were considered differentially expressed.

### qRT-PCR analysis

Liquid cultures of *Mtb* H37Rv WT and *pe25/ppe41* deletion strains were grown to early log phase in 7H9+GOTyP media and subsequently treated with 0.2 µg/mL isoniazid for 5 h. RNA was extracted using Trizol, purified, and cDNA was synthesized with SuperScript IV polymerase and random hexamer primers. mRNA expression levels for *iniB, acpM*, and *kasA* genes were quantified by qRT-PCR using SybrGreen iTaq chemistry. Expression levels were normalized to *sigA* mRNA expression, and the relative mRNA levels were calculated using the 2^−ΔΔCq^ method and plotted as the ratio of mRNA expression for each strain.

### Measuring the in vitro frequency of drug resistance

To measure the frequency of drug resistance, *Mtb H37Rv* WT and *pe25/ppe41* deletion strains were grown to an OD600 ~ 0.6 in 7H9+GOTyP medium. The cultures were diluted to approximately 1 × 10^8^ and 1 × 10^7^ CFU/mL and plated onto non-drug-containing 7H10 + GO agar plates to determine the total viable cell count, and drug-containing 7H10 agar plates supplemented with isoniazid at concentrations 10X–500X above the MIC. Plates were incubated at 37 °C for 3-4 weeks. Colonies on drug-free plates represented the total viable cell count, while colonies on drug plates indicated resistant mutants. The frequency of resistance was calculated by dividing the number of colonies on drug-containing plates by the total viable cell count from non-drug plates.

### Statistics and reproducibility

No statistical method was used to predetermine sample size. The experiments were not randomized; the Investigators were not blinded to allocation during experiments and outcome assessment. No data were excluded except in two cases: 1. The *pe_pgrs* gene family was excluded in the genetic association study due to poor mapping quality and high sequence variability. 2. ppe42 KO RNA-seq: one PPE42KO sample was excluded during quality control filtering due to minimal read recovery ( < 0.1 million reads).

### Reporting summary

Further information on research design is available in the Nature Portfolio Reporting Summary linked to this article.

## Supplementary information

Supplementary Information

Description of Additional Supplementary Files

Supplementary Data 1

Supplementary Data 2

Supplementary Data 3

Supplementary Data 4

Reporting Summary

Transparent Peer Review file

## Source data

Source Data

## Data Availability

The RNA-seq data in this study have been deposited in the NCBI Gene Expression Omnibus (GEO) database under accession code GSE302675. The data supporting the findings of this study are available within the Article and its Supplementary Information or Source Data files. Source data are provided with this paper.

## Code availability

The code used for the genetic association study (GWAS) is available in the following public repository: https://github.com/RSC-RP/2025-05_grundner_gwas_Mtub. The code used for RNA sequencing analysis is available in the following public repository https://github.com/robertdouglasmorrison/DuffyNGS and https://github.com/robertdouglasmorrison/DuffyTools.

## References

[1] Brennan, P. J. & Nikaido, H. The envelope of mycobacteria. *Annu. Rev. Biochem.* **64**, 29–63 (1995). 7574484 10.1146/annurev.bi.64.070195.000333

[2] Dulberger, C. L., Rubin, E. J. & Boutte, C. C. The mycobacterial cell envelope - a moving target. *Nat. Rev. Microbiol.* **18**, 47–59 (2020). 31728063 10.1038/s41579-019-0273-7

[3] Nikaido, H. & Jarlier, V. Permeability of the mycobacterial cell wall. *Res. Microbiol.* **142**, 437–443 (1991). 1871430 10.1016/0923-2508(91)90117-s

[4] Cole, S. T. et al. Deciphering the biology of Mycobacterium tuberculosis from the complete genome sequence. *Nature* **393**, 537–544 (1998). 9634230 10.1038/31159

[5] Babu Sait, M. R. et al. PPE51 mediates uptake of trehalose across the mycomembrane of Mycobacterium tuberculosis. *Sci. Rep.* **12**, 2097 (2022). 35136132 10.1038/s41598-022-06109-7 PMC8826857

[6] Boradia, V., Frando, A. & Grundner, C. The Mycobacterium tuberculosis PE15/PPE20 complex transports calcium across the outer membrane. *PLoS Biol.* **20**, e3001906 (2022). 36441815 10.1371/journal.pbio.3001906 PMC9731449

[7] Mitra, A., Speer, A., Lin, K., Ehrt, S. & Niederweis, M. PPE surface proteins are required for heme utilization by mycobacterium tuberculosis. *mBio* **8**, e01720–16 (2017). 28119467 10.1128/mBio.01720-16 PMC5263243

[8] Wang, Q. et al. PE/PPE proteins mediate nutrient transport across the outer membrane of Mycobacterium tuberculosis. *Science* **367**, 1147–1151 (2020). 32139546 10.1126/science.aav5912 PMC11036889

[9] Sankey, N. et al. Role of the Mycobacterium tuberculosis ESX-4 secretion system in heme iron utilization and pore formation by PPE proteins. *mSphere* **8**, e0057322 (2023). 36749044 10.1128/msphere.00573-22 PMC10117145

[10] Sarathy, J. P., Dartois, V. & Lee, E. J. The role of transport mechanisms in mycobacterium tuberculosis drug resistance and tolerance. *Pharmacology* **5**, 1210–1235 (2012). 10.3390/ph5111210 PMC3816664 24281307

[11] Laws, M., Jin, P. & Rahman, K. M. Efflux pumps in Mycobacterium tuberculosis and their inhibition to tackle antimicrobial resistance. *Trends Microbiol.* **30**, 57–68 (2022). 34052094 10.1016/j.tim.2021.05.001

[12] Walker, T. M. et al. Whole-genome sequencing for prediction of Mycobacterium tuberculosis drug susceptibility and resistance: a retrospective cohort study. *Lancet Infect. Dis.* **15**, 1193–1202 (2015). 26116186 10.1016/S1473-3099(15)00062-6 PMC4579482

[13] Rosenthal, A. et al. The TB portals: an open-access, web-based platform for global drug-resistant-tuberculosis data sharing and analysis. *J. Clin. Microbiol* **55**, 3267–3282 (2017). 28904183 10.1128/JCM.01013-17 PMC5654911

[14] The CRyPTIC Consortium Genome-wide association studies of global Mycobacterium tuberculosis resistance to 13 antimicrobials in 10,228 genomes identify new resistance mechanisms. *PLoS Biol.* **20**, e3001755 (2022). 35944070 10.1371/journal.pbio.3001755 PMC9363015

[15] Copin, R. et al. Sequence diversity in the pe_pgrs genes of Mycobacterium tuberculosis is independent of human T cell recognition. *mBio* **5**, e00960–00913 (2014). 24425732 10.1128/mBio.00960-13 PMC3903279

[16] Gomez-Gonzalez, P. J. et al. Functional genetic variation in pe/ppe genes contributes to diversity in Mycobacterium tuberculosis lineages and potential interactions with the human host. *Front. Microbiol.* **14**, 1244319 (2023). 37876785 10.3389/fmicb.2023.1244319 PMC10591178

[17] McEvoy, C. R. et al. Comparative analysis of Mycobacterium tuberculosis pe and ppe genes reveals high sequence variation and an apparent absence of selective constraints. *PLoS One* **7**, e30593 (2012). 22496726 10.1371/journal.pone.0030593 PMC3319526

[18] Marin, M. et al. Benchmarking the empirical accuracy of short-read sequencing across the M. tuberculosis genome. *Bioinformatics* **38**, 1781–1787 (2022). 35020793 10.1093/bioinformatics/btac023 PMC8963317

[19] Farhat, M. R. et al. GWAS for quantitative resistance phenotypes in Mycobacterium tuberculosis reveals resistance genes and regulatory regions. *Nat. Commun.* **10**, 2128 (2019). 31086182 10.1038/s41467-019-10110-6 PMC6513847

[20] Organization, W. H. WHO consolidated guidelines on drug-resistant tuberculosis. (WHO, 2019). 30946559

[21] Martini, M. C. et al. Loss of RNase J leads to multi-drug tolerance and accumulation of highly structured mRNA fragments in Mycobacterium tuberculosis. *PLoS Pathog.* **18**, e1010705 (2022). 35830479 10.1371/journal.ppat.1010705 PMC9312406

[22] van Kessel, J. C. & Hatfull, G. F. Recombineering in Mycobacterium tuberculosis. *Nat. Methods* **4**, 147–152 (2007). 17179933 10.1038/nmeth996

[23] Domenech, P. & Reed, M. B. Rapid and spontaneous loss of phthiocerol dimycocerosate (PDIM) from Mycobacterium tuberculosis grown in vitro: implications for virulence studies. *Microbiology* **155**, 3532–3543 (2009). 19661177 10.1099/mic.0.029199-0 PMC5154741

[24] Mulholland, C. V. et al. Propionate prevents loss of the PDIM virulence lipid in Mycobacterium tuberculosis. *Nat. Microbiol.* **9**, 1607–1618 (2024). 38740932 10.1038/s41564-024-01697-8 PMC11253637

[25] Mailaender, C. et al. The MspA porin promotes growth and increases antibiotic susceptibility of both Mycobacterium bovis BCG and Mycobacterium tuberculosis. *Microbiology* **150**, 853–864 (2004). 15073295 10.1099/mic.0.26902-0

[26] Nebenzahl-Guimaraes, H., Jacobson, K. R., Farhat, M. R. & Murray, M. B. Systematic review of allelic exchange experiments aimed at identifying mutations that confer drug resistance in Mycobacterium tuberculosis. *J. Antimicrob. Chemother.* **69**, 331–342 (2014). 24055765 10.1093/jac/dkt358 PMC3886931

[27] Safi, H. et al. Evolution of high-level ethambutol-resistant tuberculosis through interacting mutations in decaprenylphosphoryl-beta-D-arabinose biosynthetic and utilization pathway genes. *Nat. Genet* **45**, 1190–1197 (2013). 23995136 10.1038/ng.2743 PMC6103293

[28] Wilson, M. et al. Exploring drug-induced alterations in gene expression in Mycobacterium tuberculosis by microarray hybridization. *Proc. Natl. Acad. Sci. USA* **96**, 12833–12838 (1999). 10536008 10.1073/pnas.96.22.12833 PMC23119

[29] Perdigao, J. et al. Genetic analysis of extensively drug-resistant Mycobacterium tuberculosis strains in Lisbon, Portugal. *J. Antimicrob. Chemother.* **65**, 224–227 (2010). 20028780 10.1093/jac/dkp452

[30] Perdigao, J. et al. Unraveling Mycobacterium tuberculosis genomic diversity and evolution in Lisbon, Portugal, a highly drug resistant setting. *BMC Genomics* **15**, 991 (2014). 25407810 10.1186/1471-2164-15-991 PMC4289236

[31] Portugal, I. et al. Outbreak of multiple drug-resistant tuberculosis in Lisbon: detection by restriction fragment length polymorphism analysis. *Int J. Tuberc. Lung Dis.* **3**, 207–213 (1999). 10094321

[32] Machado, D. et al. High-level resistance to isoniazid and ethionamide in multidrug-resistant Mycobacterium tuberculosis of the Lisboa family is associated with inhA double mutations. *J. Antimicrob. Chemother.* **68**, 1728–1732 (2013). 23539241 10.1093/jac/dkt090

[33] Brennan, P. J. Structure, function, and biogenesis of the cell wall of Mycobacterium tuberculosis. *Tuberculosis* **83**, 91–97 (2003). 12758196 10.1016/s1472-9792(02)00089-6

[34] Danilchanka, O., Pavlenok, M. & Niederweis, M. Role of porins for uptake of antibiotics by Mycobacterium smegmatis. *Antimicrob. Agents Chemother.* **52**, 3127–3134 (2008). 18559650 10.1128/AAC.00239-08 PMC2533485

[35] Stephan, J., Mailaender, C., Etienne, G., Daffe, M. & Niederweis, M. Multidrug resistance of a porin deletion mutant of Mycobacterium smegmatis. *Antimicrob. Agents Chemother.* **48**, 4163–4170 (2004). 15504836 10.1128/AAC.48.11.4163-4170.2004 PMC525411

[36] Charitou, V. et al. PPE51 modulates membrane integrity in Mycobacterium marinum. *mBio* **16**, e0104425 (2025). 40980894 10.1128/mbio.01044-25 PMC12607722

[37] Gan, M., Wang, D., Li, S., Wang, Q. & Liu, Q. Ongoing evolution of PE/PPE genes in Mycobacterium tuberculosis associated with drug resistance and host immune response. *mSystems* **10**, e0089825 (2025). 40980874 10.1128/msystems.00898-25 PMC12542625

[38] Li, S. et al. CRISPRi chemical genetics and comparative genomics identify genes mediating drug potency in Mycobacterium tuberculosis. *Nat. Microbiol.* **7**, 766–779 (2022). 35637331 10.1038/s41564-022-01130-y PMC9159947

[39] Ates, L. S. et al. Essential role of the ESX-5 secretion system in outer membrane permeability of pathogenic mycobacteria. *PLoS Genet.* **11**, e1005190 (2015). 25938982 10.1371/journal.pgen.1005190 PMC4418733

[40] Li, H. & Durbin, R. Fast and accurate short read alignment with Burrows-Wheeler transform. *Bioinformatics* **25**, 1754–1760 (2009). 19451168 10.1093/bioinformatics/btp324 PMC2705234

[41] Li, H. Minimap2: pairwise alignment for nucleotide sequences. *Bioinformatics* **34**, 3094–3100 (2018). 29750242 10.1093/bioinformatics/bty191 PMC6137996

[42] Li, H. New strategies to improve minimap2 alignment accuracy. *Bioinformatics* **37**, 4572–4574 (2021). 34623391 10.1093/bioinformatics/btab705 PMC8652018

[43] Danecek, P. et al. Twelve years of SAMtools and BCFtools. *Gigascience* **10**, giab008 (2021). 33590861 10.1093/gigascience/giab008 PMC7931819

[44] Phelan, J. E. et al. Integrating informatics tools and portable sequencing technology for rapid detection of resistance to anti-tuberculous drugs. *Genome Med* **11**, 41 (2019). 31234910 10.1186/s13073-019-0650-x PMC6591855

[45] Lees, J. A., Galardini, M., Bentley, S. D., Weiser, J. N. & Corander, J. pyseer: a comprehensive tool for microbial pangenome-wide association studies. *Bioinformatics* **34**, 4310–4312 (2018). 30535304 10.1093/bioinformatics/bty539 PMC6289128

[46] Murphy, K. C., Papavinasasundaram, K. & Sassetti, C. M. Mycobacterial recombineering. *Methods Mol. Biol.* **1285**, 177–199 (2015). 25779316 10.1007/978-1-4939-2450-9_10

[47] Culviner, P. H., Guegler, C. K. & Laub, M. T. A simple, cost-effective, and robust method for rRNA depletion in RNA-sequencing studies. *mBio* **11**, e00010–e00020 (2020). 32317317 10.1128/mBio.00010-20 PMC7175087

