# Supplementary material for: PE/PPE proteins contribute to Mycobacterium tuberculosis drug resistance
Source: Nat Commun. 2026 Apr 24;17:5668. doi: 10.1038/s41467-026-72431-7 (PMC13315784; doi:10.1038/s41467-026-72431-7)
Supplement: Supplementary file 1 — Supplementary Information [file 41467_2026_72431_MOESM1_ESM.pdf]

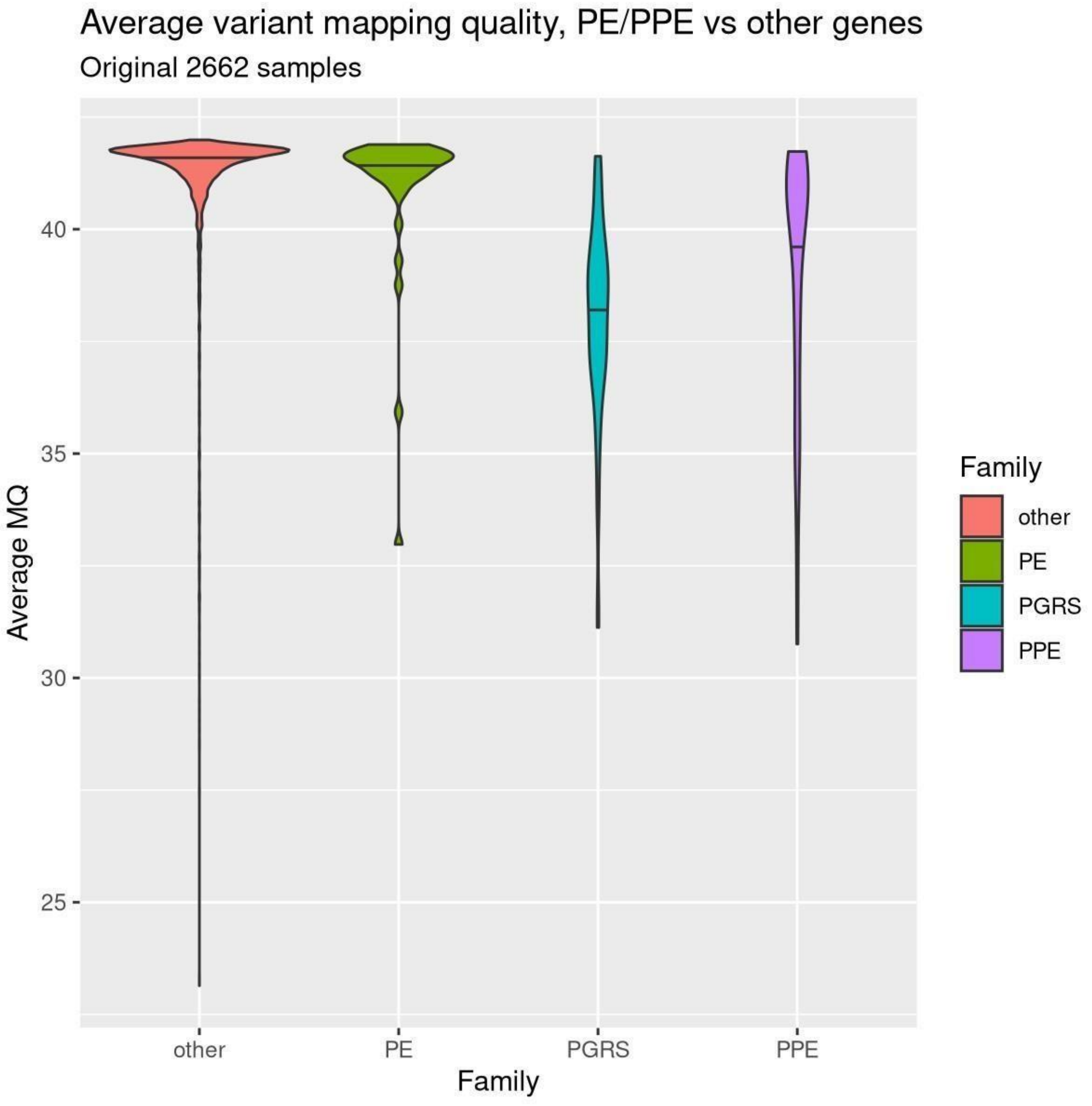

Supplementary Figure 1: Mapping quality comparison of *pe, ppe,* and *pe-pgrs* genes. Within 4,031 protein-coding genes in the *M. tuberculosis* H37Rv v3 reference genome, 35 were classified as *pe*, 69 as *ppe*, 74 as *pe-pgrs*, and 3,863 as others (x-axis). Sequences from the 2,659 strains from the Wellcome Trust Sanger Institute pilot study were aligned to the reference using Bowtie2 and genotyped using GATK HaplotypeCaller, with joint calling performed by GATK GenomicsDBImport and GenotypeGVCFs. With each gene, the mapping quality (MQ) as reported by GATK was averaged across all variants (y-axis).

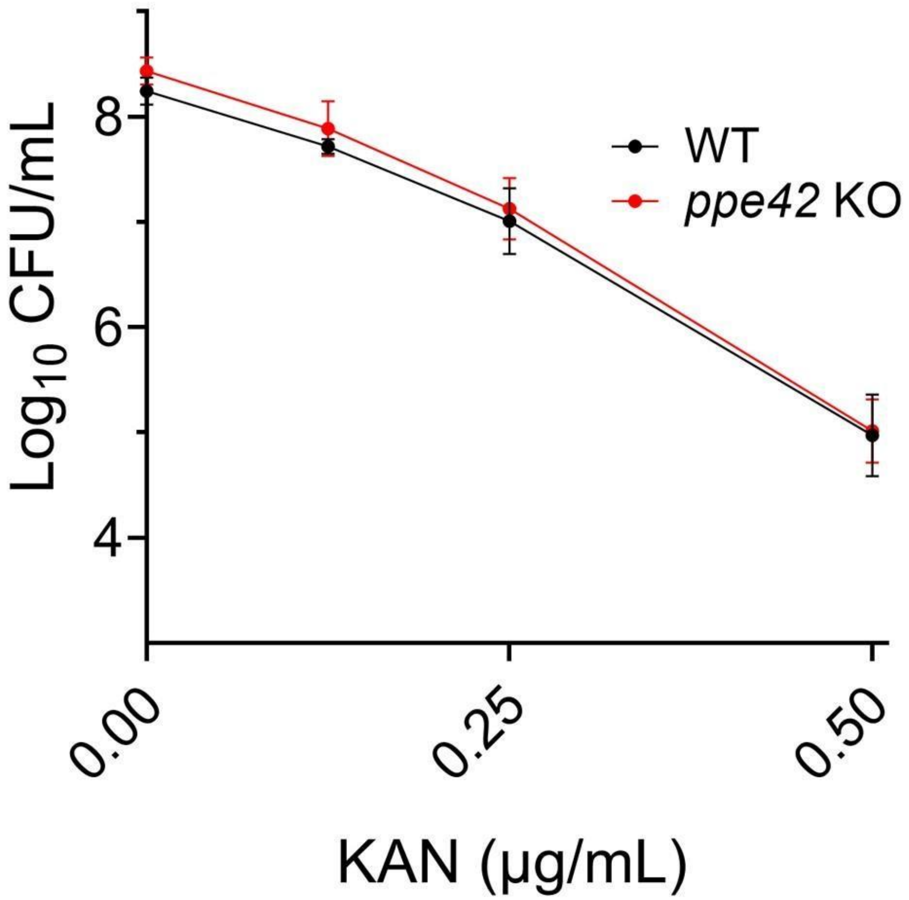

Supplementary Figure 2: CFU data for WT and *ppe42* deletion strain grown in kanamycin. Cultures were grown with drug for 7 days before spotting on agar without drug. Bacteria were enumerated by colony forming units (CFU) assay and data are shown as the mean ± SEM of four biological replicates. Statistical significance was determined by two-sided multiple comparison t-test. All the *p-value* are mentioned in supplementary data 2. Abbreviations: KAN: kanamycin

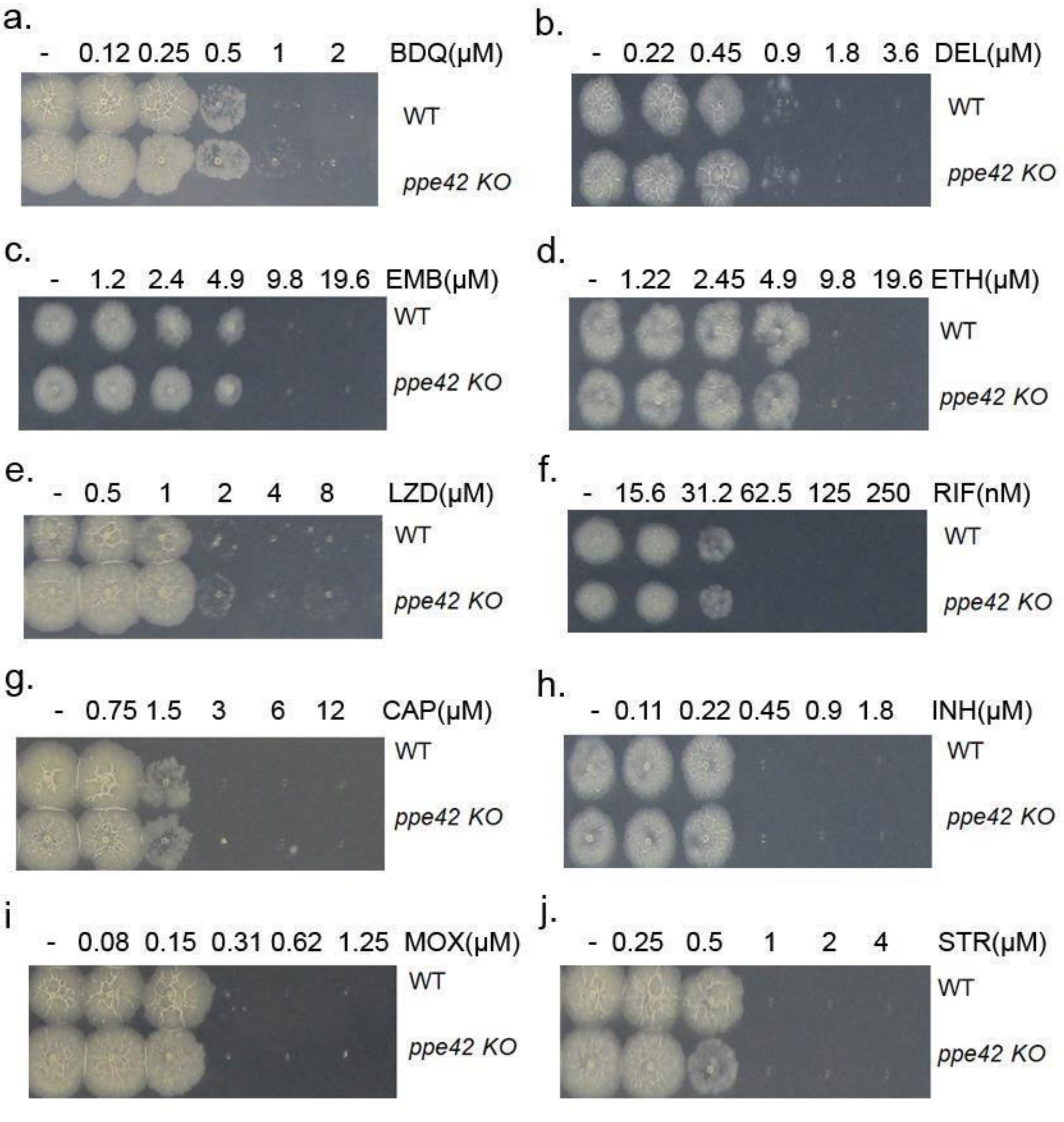

Supplementary Figure 3: Spotting data for *ppe42* deletion mutant. Cultures were grown with drug for 7 days before spotting on agar without drug. Abbreviations: BDQ: bedaquiline. DEL: delamanid. EMB: ethambutol. ETH: ethionamide. LZD: linezolid. RIF: rifampicin. CAP: capreomycin. INH: isoniazid. MOX: moxifloxacin. STR: streptomycin.

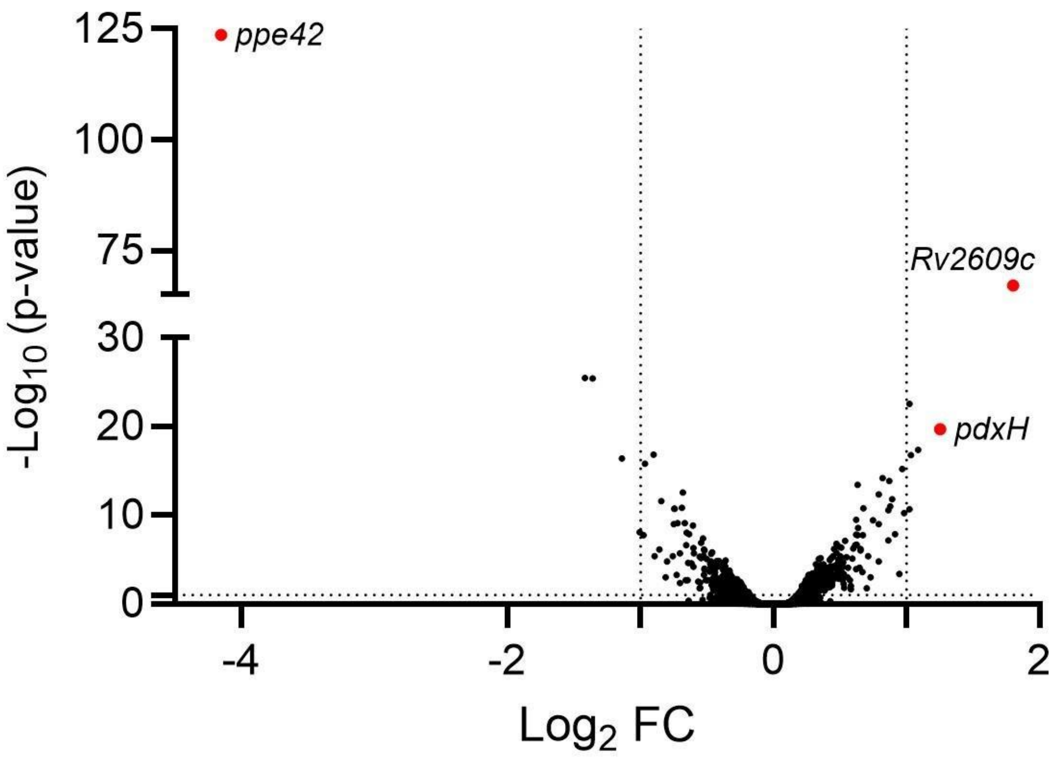

Gene	Product	Log2FC	p-value
Rv2609c	membrane protein	1.8026	8.40E-68
pdxH	pyridoxine/pyridoxamine 5'-phosphate oxidase	1.2536	2.01E-20
rpfC	resuscitation-promoting factor RpfC	1.0872	4.05E-18
Rv2254c	integral membrane protein	1.0344	1.66E-17
grpE	stress response protein GrpE	1.0225	2.23E-11
csm3	CRISPR type III-associated RAMP protein Csm3	1.022	2.86E-23
mmsA	methylmalonate-semialdehyde dehydrogenase	-1.0063	8.40E-09
fprB	ferredoxin/ferredoxin--NADP reductase	-1.1407	3.84E-17
Rv3049c	monooxygenase	-1.3585	3.74E-26
Rv0885	hypothetical protein	-1.4194	3.08E-26
ppe42	PPE family protein PPE42	-4.155	2.29E-124

Supplementary Figure 4: RNA-seq analysis of the *ppe42* deletion strain. Volcano plot shows genes differentially expressed upon *ppe42* deletion. Table shows genes with the largest fold-change compared to WT. All differentially expressed genes are given in Supplementary Data 3. Gene expression changes were identified using a combination of five differential expression tools within DuffyTools: round robin, RankProduct, significance analysis of microarrays (SAMs), EdgeR, and DeSeq2. The results from each DE tool were combined using a weighted average of fold change and significance (*p-value*).

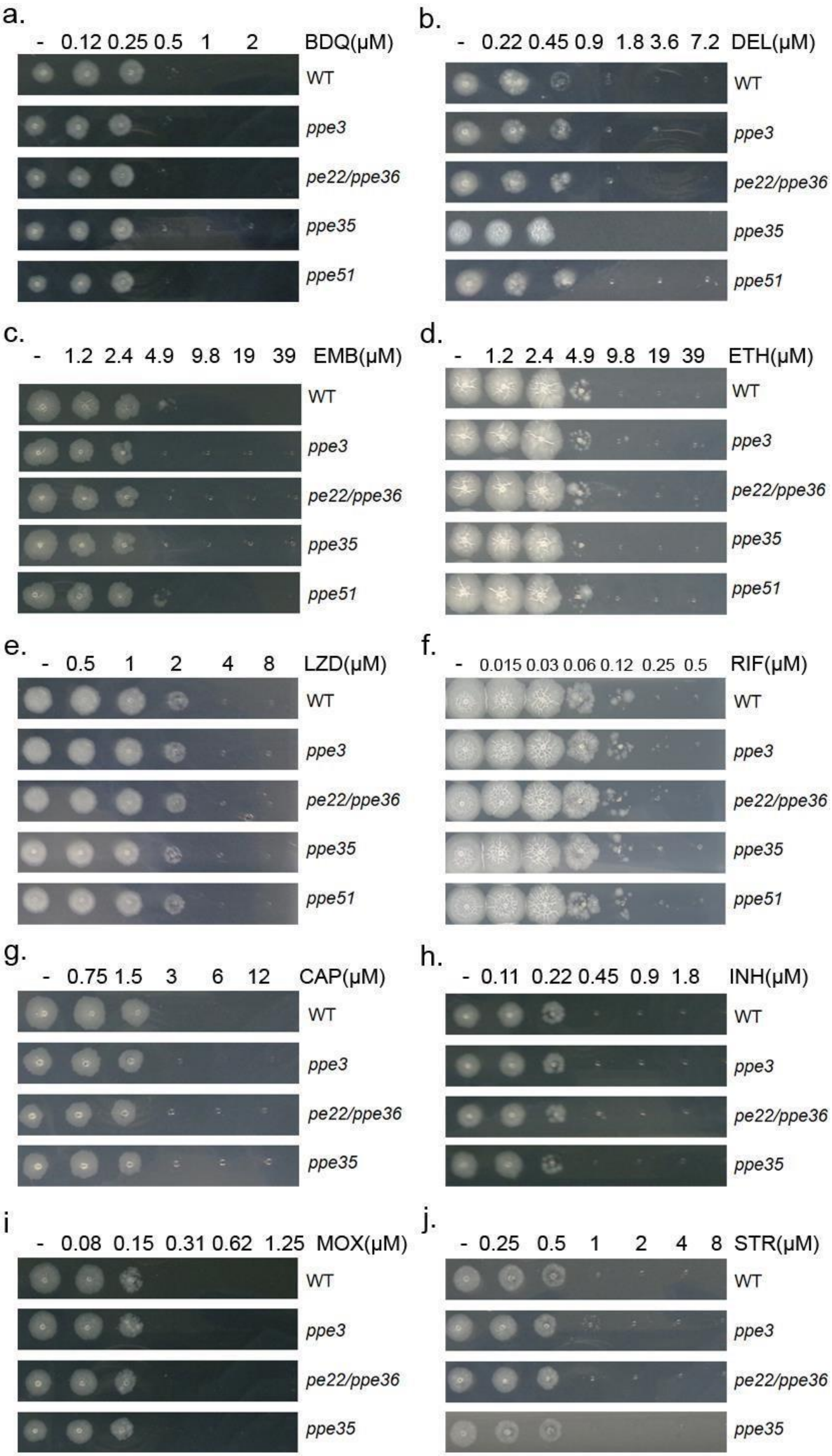
a.
- 0.12 0.25 0.5 1 2 BDQ(μM)
WT
ppe3
pe22/ppe36
ppe35
ppe51
b.
- 0.22 0.45 0.9 1.8 3.6 7.2 DEL(μM)
WT
ppe3
pe22/ppe36
ppe35
ppe51
c.
- 1.2 2.4 4.9 9.8 19 39 EMB(μM)
WT
ppe3
pe22/ppe36
ppe35
ppe51
d.
- 1.2 2.4 4.9 9.8 19 39 ETH(μM)
WT
ppe3
pe22/ppe36
ppe35
ppe51
e.
- 0.5 1 2 4 8 LZD(μM)
WT
ppe3
pe22/ppe36
ppe35
ppe51
f.
- 0.015 0.03 0.06 0.12 0.25 0.5 RIF(μM)
WT
ppe3
pe22/ppe36
ppe35
ppe51
g.
- 0.75 1.5 3 6 12 CAP(μM)
WT
ppe3
pe22/ppe36
ppe35
h.
- 0.11 0.22 0.45 0.9 1.8 INH(μM)
WT
ppe3
pe22/ppe36
ppe35
i
- 0.08 0.15 0.31 0.62 1.25 MOX(μM)
WT
ppe3
pe22/ppe36
ppe35
j.
- 0.25 0.5 1 2 4 8 STR(μM)
WT
ppe3
pe22/ppe36
ppe35

Supplementary Figure 5: Spotting data for hits from genetic association study. Cultures were grown with drug for 7 days before spotting on agar without drug. Abbreviations: BDQ: bedaquiline. DEL: delamanid. EMB: ethambutol. ETH: ethionamide. LZD: linezolid. RIF: rifampicin. CAP: capreomycin. INH: isoniazid. MOX: moxifloxacin. STR: streptomycin.

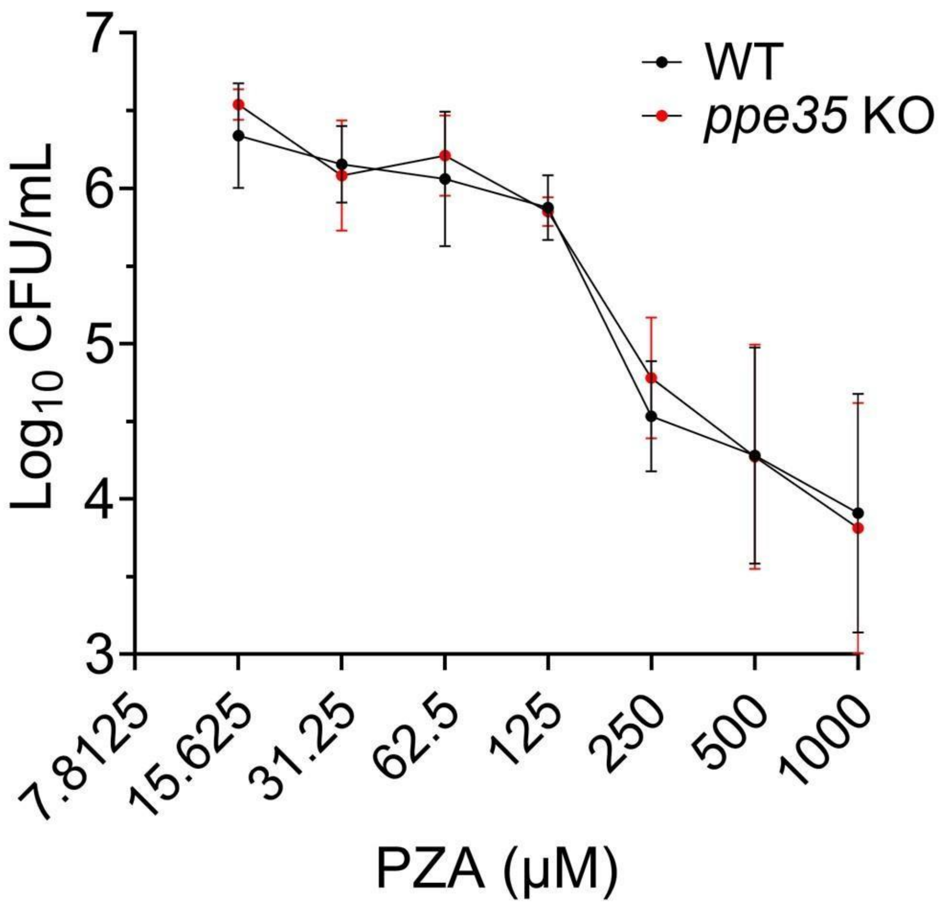

Supplementary Figure 6: CFU data for WT and *ppe35* deletion mutant grown in pyrazinamide. Cultures were grown with drug for 7 days before spotting on agar without drug. Bacteria were enumerated by colony forming units (CFU) assay and data are shown as the mean ± SD of four biological replicates. Statistical significance was determined by two-sided multiple comparison t-test. All the *p-value* are mentioned in supplementary data 2. Abbreviations: PZA: pyrazinamide

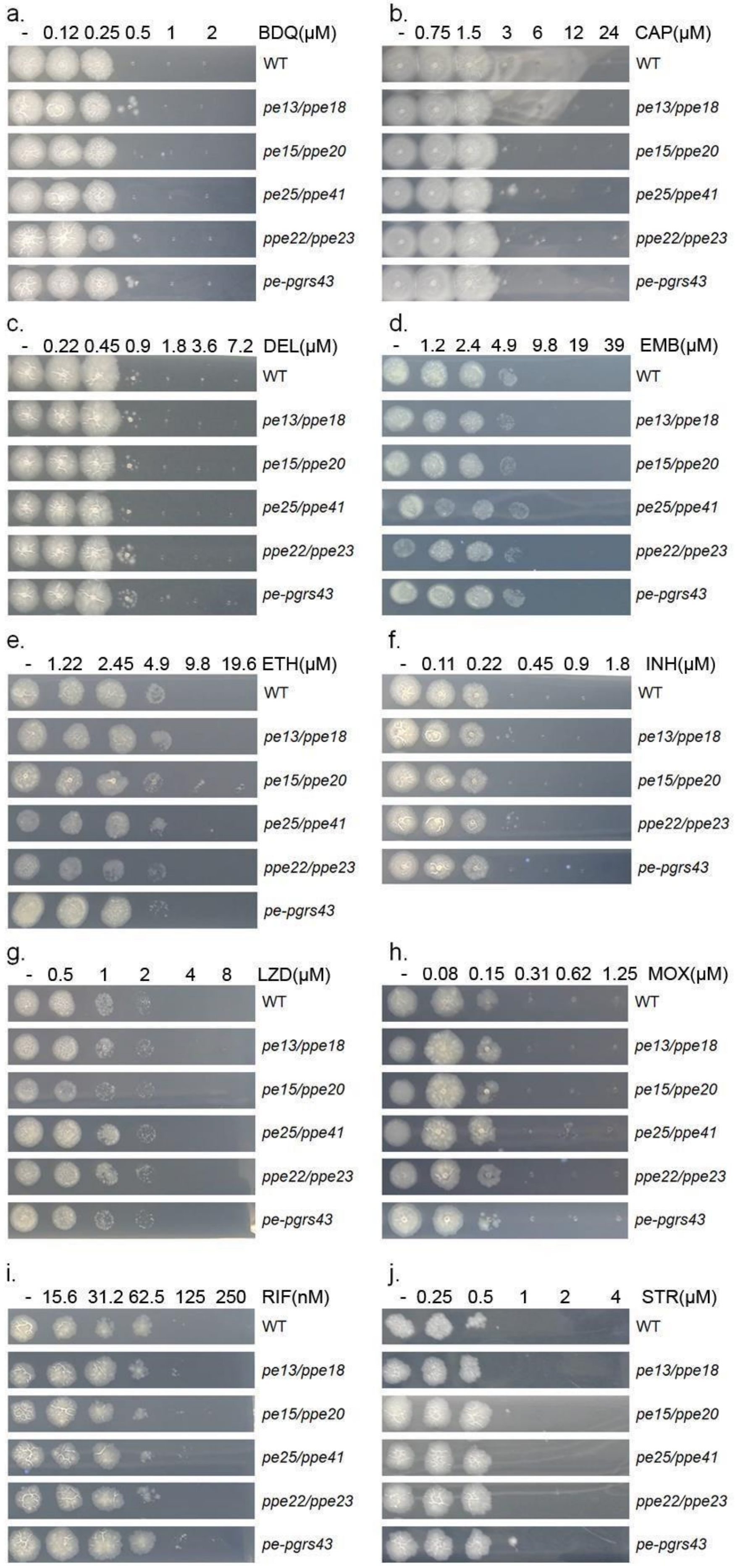
a.
- 0.12 0.25 0.5 1 2 BDQ(μM)
b.
- 0.75 1.5 3 6 12 24 CAP(μM)
c.
- 0.22 0.45 0.9 1.8 3.6 7.2 DEL(μM)
d.
- 1.2 2.4 4.9 9.8 19 39 EMB(μM)
e.
- 1.22 2.45 4.9 9.8 19.6 ETH(μM)
f.
- 0.11 0.22 0.45 0.9 1.8 INH(μM)
g.
- 0.5 1 2 4 8 LZD(μM)
h.
- 0.08 0.15 0.31 0.62 1.25 MOX(μM)
i.
- 15.6 31.2 62.5 125 250 RIF(nM)
j.
- 0.25 0.5 1 2 4 STR(μM)
WT
pe13/ppe18
pe15/ppe20
pe25/ppe41
ppe22/ppe23
pe-pgrs43

Supplementary Figure 7: Spotting data for candidates from the RNA-seq analysis. Cultures were grown with drug for 7 days before spotting on agar without drug. Abbreviations: BDQ: bedaquiline. DEL: delamanid. EMB: ethambutol. ETH: ethionamide. LZD: linezolid. RIF: rifampicin. CAP: capreomycin. INH: isoniazid. MOX: moxifloxacin. STR: streptomycin.

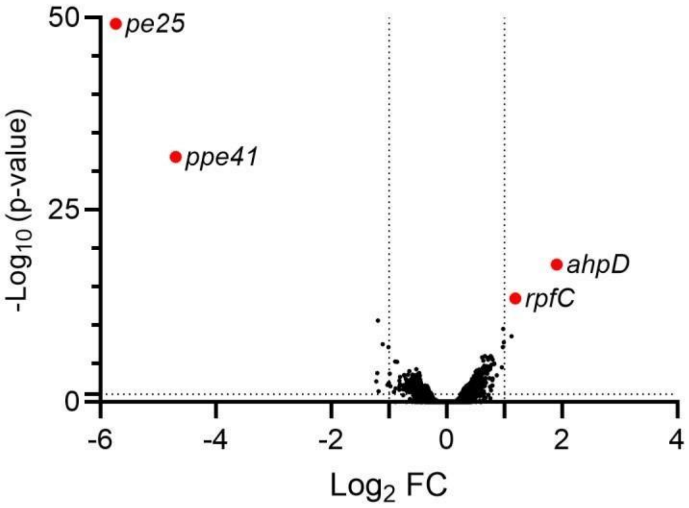

Gene	Product	Log₂FC	p-value
ahpD	alkyl hydroperoxide reductase AphD	1.9081	1.31E-18
rpfC	resuscitation-promoting factor RpfC	1.1919	3.30E-14
Rv0990c	hypothetical protein	1.1244	2.83E-09
Rv1577c	phage prohead protease	-1.0008	3.22E-03
Rv1372	alpha-pyrone synthesis polyketide synthase-like protein	-1.0106	6.86E-08
Rv2650c	prophage protein	-1.0255	5.92E-03
cas2	CRISPR-associated endoribonuclease Cas2	-1.1107	2.97E-08
Rv3126c	hypothetical protein	-1.1788	3.86E-02
moeY	molybdopterin biosynthesis protein MoeY	-1.1897	2.47E-11
Rv2087	conserved hypothetical protein	-1.2053	1.70E-04
desA3	stearoyl-CoA 9-desaturase	-1.2207	2.08E-03
ppe41	PPE family protein PPE41	-4.6962	1.27E-32
pe25	PE family protein PE25	-5.7362	5.83E-50

Supplementary Figure 8: RNA-seq analysis of the *pe25/ppe41* deletion strain. Volcano plot shows differentially expressed genes upon *pe25/ppe41* deletion. Table shows genes with the largest fold-change compared to WT. All differentially expressed genes are given in Supplementary Data 3. Gene expression changes were identified using a combination of five differential expression tools within DuffyTools: round robin, RankProduct, significance analysis of microarrays (SAMs), EdgeR, and DeSeq2. The results from each DE tool were combined using a weighted average of fold change and significance (*p-value*).

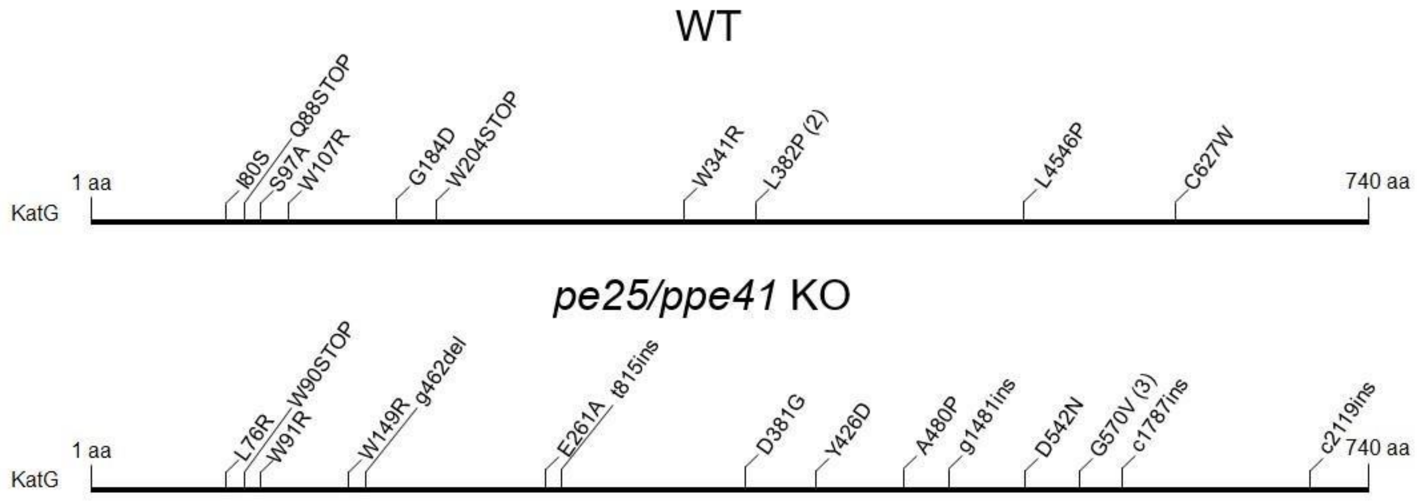

Supplementary Figure 9: *katG* mutations in INH-resistant clones of the *pe25/ppe41* deletion strain. Mutations were identified by DNA sequencing of the *katG* gene. The corresponding amino acid changes in KatG are shown. Numbers in parentheses indicate multiple occurrences of the mutation.

Supplementary Table 1: Genes related to PDIM biosynthesis tested by RNA-seq.

List of genes tested for mutations related to PDIM in the RNA seq using DuffyNGS pipi.PlotSNP() script*	
Phthiocerol synthesis (PpsABCDE): Modular polyketide synthases (PKS)	*ppsA (Rv2931)*
	ppsB (Rv2932)
	ppsC (Rv2933)
	ppsD (Rv2934)
	ppsE (Rv2935)
Mycocerosic acid synthesis	*mas (Rv2940c)*
	fadD28 (Rv2941)
Regulation and transport	*fadD26 (Rv2930)*
	mmpL7 (Rv2942)
	drrABC (Rv2936–Rv2938)
	papA5 (Rv2939)

*https://github.com/robertdouglasmorrison/DuffyNGS
