# Supplementary material for: PE/PPE proteins contribute to Mycobacterium tuberculosis drug resistance
Source: Nat Commun. 2026 Apr 24;17:5668. doi: 10.1038/s41467-026-72431-7 (PMC13315784; doi:10.1038/s41467-026-72431-7)
Supplement: Supplementary file 2 — Description of Additional Supplementary Files [file 41467_2026_72431_MOESM2_ESM.pdf]

Description of Additional Supplementary Files

File Name: Supplementary Data 1
Description: Genetic associations between clinical drug resistance and *pe* and *ppe* genes. All associations from our targeted association study with an FDR below 10^{-5} are listed.

File Name: Supplementary Data 2
Description: *p*-values for all the graphs.

File Name: Supplementary Data 3
Description: Differentially expressed genes in the *pe/ppe* deletion strains compared to WT. RNA-seq analysis was done after four days of growth.

File Name: Supplementary Data 4
Description: Primers used for generating knockout strains, complementation strains, and primers used for qRT-PCR. Source data for Figure 3a.
