# Supplementary material for: PE/PPE proteins contribute to Mycobacterium tuberculosis drug resistance
Source: Nat Commun. 2026 Apr 24;17:5668. doi: 10.1038/s41467-026-72431-7 (PMC13315784; doi:10.1038/s41467-026-72431-7)
Supplement: Supplementary file 8 — Transparent Peer Review file [file 41467_2026_72431_MOESM8_ESM.pdf]

nature portfolio

Peer Review File

PE/PPE proteins contribute to Mycobacterium tuberculosis drug resistance

Corresponding Author: Professor Christoph Grundner

This file contains all reviewer reports in order by version, followed by all author rebuttals in order by version.

Version 0:

Reviewer comments:

Reviewer #1

(Remarks to the Author)
In this manuscript, Boradia et al. bring together disparate observations and hypotheses from the literature to test the idea that PE/PPE proteins contribute to antibiotic tolerance and to the development of resistance in Mycobacterium tuberculosis. This work is significant from a biological perspective because M. tuberculosis, in contrast to Gram-negatives and the model organism M. smegmatis, lacks obvious porin-like proteins. This work is also significant from a medical perspective because genes that promote drug uptake may serve as predictive biomarkers for developing drug resistance. The experiments comprise a rigorous mix of clinical and lab-generated data. My suggestions primarily pertain to framing and context.

BIG PICTURE

The authors do not directly test the underlying premise of their work, which is that the PE/PPEs are conduits for drug uptake. Except for Fig. 3f -- a clever use of gene expression to read out isoniazid uptake -- they posit that differences in antibiotic susceptibility are proxies for differences in uptake. This is not a problem per se as the manuscript is, in general, carefully written. However, it is framed (Introduction/Discussion) around the idea that PE/PPEs are MOM channels. This is a very exciting idea, and consistent with the data, but there is no direct evidence, for example, mass spec of drug uptake in bacteria +/- pe/ppe gene of interest or in vitro experiments to assess channel formation by purified proteins. Because such studies are beyond the scope of the current work, I would simply recommend more nuanced writing:

• Lines 142-143 and 198-200: MspA reversion experiments are solid but there are other interpretations; for example, heterologous expression of MspA might alter the MOM structure and impact uptake in a manner that is distinct from transit through an aqueous channel. For example PMID 15504836 and PMID 18559650.

• Introduction/Discussion: by focusing on PE/PPEs as potential MOM conduits, the authors may be doing themselves a disservice. First, because of the reasons I cite above, but perhaps equally importantly, a real strength of their work is merging clinical and lab-generated data. Framing would also be strengthened by additional context: a) PMID 32138343 reports that ppe51 mutants have no difference in susceptibility to multiple antibiotics b) PMID 40980874 and PMID 37352142 (perhaps others?) show conceptually-similar analyses to Fig. 1a c) PMID 40980894 and PMID 32139546 speak to secondary effects related to loss of ppe51 that may pertain to interpretation of Fig. 2 including point made in a). Several of these papers were very recently published, so it is reasonable that they were not included in the initial draft, but it would benefit the manuscript and the field to include them in a revision.

DETAILS

• Lines 50-51: This sentence should be reworked. “As a result” implies that we know why there is a difference in mycobacteria vs. Gram-negatives. Given the dramatic differences in OM structure between these bacteria, I’m not sure we can yet attribute everything to porin-like proteins or lack thereof, especially given that the references cited only queried a handful of molecules.
• Lines 62-63 can this be referenced or is it an observation made by the authors?
• Lines 66-69 and 158-159: efflux is more controversial than written, e.g. PMID 34637511. Related, does the spectrum of antibiotics that are impacted by MmpL5/Mmps5 and/or Rv1216c-1218c and/or Mmr in the literature match what the authors observe for the ppe51 mutant?

• Lines 146 and 151: “increased susceptibility” I believe is a typo
• It would be helpful for the authors to include antibiotic concentrations used in spotting in the figures, so that readers can more easily compare to the fixed concentration experiments.

Reviewer #2

(Remarks to the Author)
I co-reviewed this manuscript with one of the reviewers who provided the listed reports. This is part of the Nature Communications initiative to facilitate training in peer review and to provide appropriate recognition for Early Career Researchers who co-review manuscripts.

Reviewer #3

(Remarks to the Author)
This is a highly interesting and potentially impactful manuscript. The authors have performed some careful and well crafted work to demonstrate the role of some of the PE/PPE genes in drug susceptibility. There is much more work to be done in the space, but this manuscript very effectively opens up this corner of the field for future endeavour.
My only minor comments is that there looks to be a typo in line 150 where it says "The deletion
mutant showed increased susceptibility to amikacin, capreomycin, moxifloxacin, streptomycin, and INH (Fig. 2a)"
I think the authors probably mean decreased susceptibility here as the KO strain grows at higher concentrations in fig 2a, which would indicate lower susceptibility to the drug.

Reviewer #4

(Remarks to the Author)
I co-reviewed this manuscript with one of the reviewers who provided the listed reports. This is part of the Nature Communications initiative to facilitate training in peer review and to provide appropriate recognition for Early Career Researchers who co-review manuscripts.

Reviewer #5

(Remarks to the Author)
This manuscript explores how selected PE/PPE proteins modulate antimycobacterial drug responses. The study combines genome-wide association, targeted knockouts, and mechanistic readouts to suggest that specific PE/PPEs shape susceptibility via outer-membrane transport and/or downstream efflux. The work is careful overall and potentially impactful. While several drug effects are modest, the dataset is solid and the questions are important.

Major comments:
Please include complementation of Δppe51 with WT ppe51 and the A222T clinical allele. This will both establish causality and directly validate the sequencing data.

Efflux vs uptake: If the conclusion is that PPE51 does not directly mediate uptake but rather induces inner-membrane efflux, this should be supported by simple experiments; also including ppe41/42 as controls.
- MICs/time-kill +/- efflux inhibitors (verapamil, reserpine, CCCP).
- Or EtBr accumulation/efflux kinetics as a quick functional proxy.
- Or RNAseq of Δppe42 and Δppe41 for comparison to PPE51 mutant.

Minor comments:
Because PDIM status can alter permeability and drug response, please state consistently whether mutants were maintained with propionate supplementation and provide empirical PDIM checks (or sequencing of the PDIM synthesis cluster) in the strains used for susceptibility assays.

Line 151: “The mutant showed decreased not increased susceptibility.

Line 158–160: change: effect … on drug susceptibility is likely indirect to “effect … on drug resistance is likely indirect. Add a sentence making clear, that the authors believe that the deletion of PPE51 is causing upregulation of inner membrane transporters resulting in higher drug resistance

Version 1:

Reviewer comments:

Reviewer #1

(Remarks to the Author)

The authors have done an excellent job responding to the reviews. We have a few minor points for consideration:

• Lines 151-152: "consistent with ppe42 having a similar transport function to mspA" -> suggest slight rephrasing to reflect nuance that is (well-) captured in Discussion

• Line 299: similar comment to above; suggest slight rephrasing to encompass nuance for PPE51

• Lines 278-279: please specify pe/ppe

• Methods indicate 7H9 with Tween 80 (7H9+GOT) but it appears only 7H9 with Tyloxapol (7H9+GOTyp) was used for the experiments described.

Reviewer #2

(Remarks to the Author)
I co-reviewed this manuscript with one of the reviewers who provided the listed reports. This is part of the Nature Communications initiative to facilitate training in peer review and to provide appropriate recognition for Early Career Researchers who co-review manuscripts.

Reviewer #3

(Remarks to the Author)
My small query has been addressed

Reviewer #4

(Remarks to the Author)
I co-reviewed this manuscript with one of the reviewers who provided the listed reports. This is part of the Nature Communications initiative to facilitate training in peer review and to provide appropriate recognition for Early Career Researchers who co-review manuscripts.

Reviewer #5

(Remarks to the Author)
I would like to thank the authors for their detailed response and for addressing the suggestions made during the first round of review. The manuscript has improved as a result.

Major Points:
While the authors provide a sound discussion regarding the complexity of efflux and permeability effects in the absence of PPE51, the complementation data, specifically concerning antibiotic susceptibility, remain only partial. The authors acknowledge this fact convincingly in the revised manuscript.
Given that complementation is a fundamental requirement for validating genetic phenotypes, these data are too important to be kept in the supplement. I strongly suggest moving the complementation results from the supplement into the main Figure 2.
Furthermore, I acknowledge the effort in constructing the A222T complementation strain. However, because the wild-type (WT) complementation is minimal, the current conclusion is overstating the results:
“Although complementation with the Ala222Thr mutant phenocopied the deletion strain with all five drugs, suggesting it is causal for clinical drug resistance” (Lines 319-321).
Since the WT does not fully restore the phenotype, we cannot definitively conclude that the mutant "phenocopies" the deletion in a way that proves causality. Please revise this section to reflect the limitations of the complementation data. The A222T data itself should stay in the manuscript, but may remain in the supplement if preferred.

Minor points:
Regarding the sequencing data used to verify the integrity of the PDIM synthesis cluster, which includes genes rv2930-rv2942. The manuscript should state that only minor changes in expression levels were detected. To ensure transparency and allow readers to independently verify the absence of point mutations in these genes, the raw sequencing data should be deposited and made available upon publication.

Open Access This Peer Review File is licensed under a Creative Commons Attribution 4.0 International License, which permits use, sharing, adaptation, distribution and reproduction in any medium or format, as long as you give appropriate credit to the original author(s) and the source, provide a link to the Creative Commons license, and indicate if changes were made.
In cases where reviewers are anonymous, credit should be given to 'Anonymous Referee' and the source.
The images or other third party material in this Peer Review File are included in the article's Creative Commons license, unless indicated otherwise in a credit line to the material. If material is not included in the article's Creative Commons license and your intended use is not permitted by statutory regulation or exceeds the permitted use, you will need to obtain permission directly from the copyright holder.
To view a copy of this license, visit https://creativecommons.org/licenses/by/4.0/

Response to Reviewers

We thank the reviewers for their time and effort and the very positive and thoughtful comments. Please find a point-by-point response and explanation of new data below. Changes in the revised manuscript are in blue.

Reviewer #1 (Remarks to the Author):

In this manuscript, Boradia et al. bring together disparate observations and hypotheses from the literature to test the idea that PE/PPE proteins contribute to antibiotic tolerance and to the development of resistance in Mycobacterium tuberculosis. This work is significant from a biological perspective because M. tuberculosis, in contrast to Gram-negatives and the model organism M. smegmatis, lacks obvious porin-like proteins. This work is also significant from a medical perspective because genes that promote drug uptake may serve as predictive biomarkers for developing drug resistance. The experiments comprise a rigorous mix of clinical and lab-generated data. My suggestions primarily pertain to framing and context.

BIG PICTURE

The authors do not directly test the underlying premise of their work, which is that the PE/PPEs are conduits for drug uptake. Except for Fig. 3f -- a clever use of gene expression to read out isoniazid uptake -- they posit that differences in antibiotic susceptibility are proxies for differences in uptake. This is not a problem per se as the manuscript is, in general, carefully written. However, it is framed (Introduction/Discussion) around the idea that PE/PPEs are MOM channels. This is a very exciting idea, and consistent with the data, but there is no direct evidence, for example, mass spec of drug uptake in bacteria +/- pe/ppe gene of interest or in vitro experiments to assess channel formation by purified proteins. Because such studies are beyond the scope of the current work, I would simply recommend more nuanced writing:

• Lines 142-143 and 198-200: MspA reversion experiments are solid but there are other interpretations; for example, heterologous expression of MspA might alter the MOM structure and impact uptake in a manner that is distinct from transit through an aqueous channel. For example PMID 15504836 and PMID 18559650.

We agree that the mspA complementation experiments are not definitive and could be due to a more general OM effect. We discussed this possibility and added the two suggested references in the Discussion, line 305-310.

• Introduction/Discussion: by focusing on PE/PPEs as potential MOM conduits, the authors may be doing themselves a disservice. First, because of the reasons I cite above, but perhaps equally importantly, a real strength of their work is merging clinical and lab-generated data. Framing would also be strengthened by additional context: a) PMID

32138343 reports that ppe51 mutants have no difference in susceptibility to multiple antibiotics b) PMID 40980874 and PMID 37352142 (perhaps others?) show conceptually-similar analyses to Fig. 1a c) PMID 40980894 and PMID 32139546 speak to secondary effects related to loss of ppe51 that may pertain to interpretation of Fig. 2 including point made in a). Several of these papers were very recently published, so it is reasonable that they were not included in the initial draft, but it would benefit the manuscript and the field to include them in a revision.

We agree that especially considering publications since our submission, additional context is useful. We have added a section discussing recent publications and others indicated by the reviewer and thank the reviewer for pointing these out (line 311-322). Also, we tried to describe the connection between PE/PPE and OM transport in a more careful way. We chose not to cite PMID 32138343 because it only tests drug susceptibility of four point mutations in ppe51. We don't think these data are particularly conclusive for a general role of ppe51 in drug resistance.

DETAILS

• Lines 50-51: This sentence should be reworked. "As a result" implies that we know why there is a difference in mycobacteria vs. Gram-negatives. Given the dramatic differences in OM structure between these bacteria, I'm not sure we can yet attribute everything to porin-like proteins or lack thereof, especially given that the references cited only queried a handful of molecules.

Agreed, we changed "As a result" to "likely contributes"

• Lines 62-63 can this be referenced or is it an observation made by the authors?

This observation was initially reported in Ref. 8 for PPE proteins, and we added PE proteins in our own analysis. We included the reference to 8.

• Lines 66-69 and 158-159: efflux is more controversial than written, e.g. PMID 34637511. Related, does the spectrum of antibiotics that are impacted by MmpL5/Mmps5 and/or Rv1216c-1218c and/or Mmr in the literature match what the authors observe for the ppe51 mutant?

Rv1216c-1218c and Mmr have been linked to INH resistance, but the existing literature does not explain the increase in resistance to all five drugs that we observed in the ppe51 deletion strain. We agree with the reviewer that efflux is probably more complicated than generally portrayed, and the substrate spectrum for many pumps is likely only incompletely defined. We changed the statement to acknowledge these uncertainties from "the effect of ppe51 deletion on drug susceptibility is likely indirect" to "may be indirect" (line 172).

• Lines 146 and 151: “increased susceptibility” I believe is a typo

Corrected

• It would be helpful for the authors to include antibiotic concentrations used in spotting in the figures, so that readers can more easily compare to the fixed concentration experiments.

Agreed, we added drug concentrations in all relevant Figures.

Reviewer #2 (Remarks to the Author):

I co-reviewed this manuscript with one of the reviewers who provided the listed reports. This is part of the Nature Communications initiative to facilitate training in peer review and to provide appropriate recognition for Early Career Researchers who co-review manuscripts.

Reviewer #3 (Remarks to the Author):

This is a highly interesting and potentially impactful manuscript. The authors have performed some careful and well crafted work to demonstrate the role of some of the PE/PPE genes in drug susceptibility. There is much more work to be done in the space, but this manuscript very effectively opens up this corner of the field for future endeavour.
My only minor comments is that there looks to be a typo in line 150 where it says "The deletion mutant showed increased susceptibility to amikacin, capreomycin, moxifloxacin, streptomycin, and INH (Fig. 2a)". I think the authors probably mean decreased susceptibility here as the KO strain grows at higher concentrations in fig 2a, which would indicate lower susceptibility to the drug.

Thanks, corrected.

Reviewer #4 (Remarks to the Author):

I co-reviewed this manuscript with one of the reviewers who provided the listed reports. This is part of the Nature Communications initiative to facilitate training in peer review and to provide appropriate recognition for Early Career Researchers who co-review manuscripts.

Reviewer #5 (Remarks to the Author):

This manuscript explores how selected PE/PPE proteins modulate antimycobacterial drug

responses. The study combines genome-wide association, targeted knockouts, and mechanistic readouts to suggest that specific PE/PPEs shape susceptibility via outer-membrane transport and/or downstream efflux. The work is careful overall and potentially impactful. While several drug effects are modest, the dataset is solid and the questions are important.

Major comments:
Please include complementation of Δppe51 with WT ppe51 and the A222T clinical allele. This will both establish causality and directly validate the sequencing data.

We have now complemented the Δppe51 strain with WT ppe51. This strain showed partial complementation with two drugs, but not the three others that the Δppe51 strain showed higher resistance to. Given the complexities of ppe51 also affecting efflux and likely general OM permeability, this result is perhaps not surprising. Especially an OM alteration that arises from ppe51 deletion might just not be readily repaired even if ppe51 is reintroduced.

We also generated the Ala222Thr complementation strain, which behaved the same as the ppe51 deletion strain in all cases, suggesting that Ala222Thr is indeed associated with drug resistance. However, although for two drugs, both mutants behave just as expected, with the inconsistent complementation seen with WT ppe51, we cannot rule out that some of the same issues affect the results of Ala222Thr complementation. We included these new data as Fig. S5 and included a section discussing the complexities around ppe51 (lines 311-324).

Efflux vs uptake: If the conclusion is that PPE51 does not directly mediate uptake but rather induces inner-membrane efflux, this should be supported by simple experiments; also including ppe41/42 as controls.
- MICs/time-kill +/- efflux inhibitors (verapamil, reserpine, CCCP).
- Or EtBr accumulation/efflux kinetics as a quick functional proxy.
- Or RNAseq of Δppe42 and Δppe41 for comparison to PPE51 mutant.

Agreed. As suggested, we now also analyzed the ppe42 and pe25/ppe41 deletion mutant strains by RNA-seq and included the data as Figs. S4 and S9. Unlike deletion of ppe51, deletion of ppe42 and pe25/ppe41 caused only minimal overall changes in gene expression and no significant changes in efflux pumps, consistent with a unique connection between inner membrane efflux and ppe51. We clarified that our ppe51 data are consistent with a combination of altered efflux and/or uptake, but that the relative contributions are unknown at this point (lines 311-324).

Minor comments:
Because PDIM status can alter permeability and drug response, please state consistently whether mutants were maintained with propionate supplementation and provide empirical

PDIM checks (or sequencing of the PDIM synthesis cluster) in the strains used for susceptibility assays.

We added mention of propionate supplementation throughout. We also checked RNA-seq data for all WT and deletion strains used. None of the strains showed mutation or altered levels of transcription of the PDIM gene cluster or other genes involved in PDIM biosynthesis, regulation, or transport such as fadD26, mmpL7, drrABC, papA5, and others. We added a sentence explaining the sequencing analysis (lines 126-129 and Table S2). For completeness, we also added testing of the ppe42 deletion mutant against the other drugs in our panel (Fig. S3). Except for amikacin, the ppe42 KO did not show altered resistance to any other drug, indicating selectivity of this ppe. The change in resistance to only one of the ten drugs tested also indicates that there are no inadvertent PDIM changes in the ppe42 and pe25/ppe41 deletion strains.

Line 151: "The mutant showed decreased not increased susceptibility.

Corrected

Line 158–160: change: effect ... on drug susceptibility is likely indirect to "effect ... on drug resistance is likely indirect. Add a sentence making clear, that the authors believe that the deletion of PPE51 is causing upregulation of inner membrane transporters resulting in higher drug resistance

Please see lines 172-173 and 322-324.

Thanks again.

Response to reviewers:

We would like to thank the reviewers again for an exceptionally constructive and helpful review.

Reviewer #1 (Remarks to the Author):

The authors have done an excellent job responding to the reviews. We have a few minor points for consideration:

• Lines 151-152: "consistent with ppe42 having a similar transport function to mspA" -> suggest slight rephrasing to reflect nuance that is (well-) captured in Discussion
We decided to remove this statement from the Results (Line 151), as it is interpretation rather than result and the nuance of this experiment is already covered in detail in the Discussion.

• Line 299: similar comment to above; suggest slight rephrasing to encompass nuance for PPE51
We rephrased this section more carefully (lines 297-299): "When interpreted in the context of transporter function, the effects of PPE42 on amikacin susceptibility, of PPE51 potentially on several drugs, and of PE25/PPE41 on INH susceptibility would be consistent with an importer function"

• Lines 278-279: please specify pe/ppe
Done

• Methods indicate 7H9 with Tween 80 (7H9+GOT) but it appears only 7H9 with Tyloxapol (7H9+GOTyp) was used for the experiments described.
Done

Reviewer #2 (Remarks to the Author):

I co-reviewed this manuscript with one of the reviewers who provided the listed reports. This is part of the Nature Communications initiative to facilitate training in peer review and to provide appropriate recognition for Early Career Researchers who co-review manuscripts.

Reviewer #3 (Remarks to the Author):

My small query has been addressed

Reviewer #4 (Remarks to the Author):

I co-reviewed this manuscript with one of the reviewers who provided the listed reports.

This is part of the Nature Communications initiative to facilitate training in peer review and to provide appropriate recognition for Early Career Researchers who co-review manuscripts.

Reviewer #5 (Remarks to the Author):

I would like to thank the authors for their detailed response and for addressing the suggestions made during the first round of review. The manuscript has improved as a result.

Major Points:
While the authors provide a sound discussion regarding the complexity of efflux and permeability effects in the absence of PPE51, the complementation data, specifically concerning antibiotic susceptibility, remain only partial. The authors acknowledge this fact convincingly in the revised manuscript.
Given that complementation is a fundamental requirement for validating genetic phenotypes, these data are too important to be kept in the supplement. I strongly suggest moving the complementation results from the supplement into the main Figure 2.
Agreed. We moved these data into Main Figure 2.

Furthermore, I acknowledge the effort in constructing the A222T complementation strain. However, because the wild-type (WT) complementation is minimal, the current conclusion is overstating the results:
“Although complementation with the Ala222Thr mutant phenocopied the deletion strain with all five drugs, suggesting it is causal for clinical drug resistance” (Lines 319-321).
Since the WT does not fully restore the phenotype, we cannot definitively conclude that the mutant "phenocopies" the deletion in a way that proves causality. Please revise this section to reflect the limitations of the complementation data. The A222T data itself should stay in the manuscript, but may remain in the supplement if preferred.
Agreed. We changed the section to acknowledge these issues more clearly. It now reads (lines 318-324):
Complementation of the *ppe51* deletion strain with WT and the Ala222Thr mutation were not fully consistent: Although the lack of complementation with the Ala222Thr mutant with any of the five drugs could suggest that it is causal for clinical drug resistance, the WT gene only complemented with two out of the five drugs, and not to WT levels in any of the experiments. Without full complementation with the WT *ppe51* gene, the effect of the Ala222Thr mutation in these complementation experiments remains ultimately inconclusive.

Minor points:
Regarding the sequencing data used to verify the integrity of the PDIM synthesis cluster, which includes genes rv2930-rv2942. The manuscript should state that only minor changes in expression levels were detected. To ensure transparency and allow readers to

independently verify the absence of point mutations in these genes, the raw sequencing data should be deposited and made available upon publication.
We made the suggested change (line 128); the sequencing data is available in the GEO database under accession code GSE302675.
